# Supplementary material for: Timing of adverse events in patients undergoing acute and elective hip arthroplasty surgery: a multicentre cohort study using the Global Trigger Tool
Source: BMJ Open. 2023 Jun 9;13(6):e064794. doi: 10.1136/bmjopen-2022-064794 (PMC10277118; doi:10.1136/bmjopen-2022-064794)
Supplement: Supplementary data [file bmjopen-2022-064794supp001.pdf]

Table 2 Overview of identified adverse events for acute respective elective patients during different time frames and time to occurrence, sorted by descending order of types of AEs for all patients

	**All**		**Acute**					**Elective**				
**Type of AEs**	**N (%)**	**Median time from surgery to AE (days)**	**n (%)**	**Median time from surgery to AE (days)**	**Occurred during index admission n (%)**	**Occurred within 30 days but after index n (%)**	**Occurred within 31–90 days n (%)**	**n (%)**	**Median time from surgery to AE (days)**	**Occurred during index admission n (%)**	**Occurred within 30 days but after index n (%)**	**Occurred within 31–90 days n (%)**
Dislocation of the prothesis	274 (13.1)	23	101 (10.4)	23	11 (2.3)	56 (14)	34 (36.6)	173 (15.4)	23	23 (4.8)	83 (17.8)	67 (36.6)
Pressure ulcer	189 (9)	5	143 (14.8)	5	91 (19.1)	46 (11.5)	6 (6.5)	46 (4.1)	4.5	33 (6.9)	11 (2.4)	2 (1.1)
Urinary tract infection	163 (7.8)	8	93 (9.6)	9	39 (8.2)	49 (12.3)	5 (5.4)	70 (6.2)	8	32 (6.7)	36 (7.7)	2 (1.1)
Peri-prosthetic joint infection	149 (7.1)	20	36 (3.7)	19	4 (0.8)	27 (6.8)	5 (5.4)	113 (10)	20	4 (0.8)	80 (17.2)	29 (15.8)
Superficial wound infection	147 (7)	11	59 (6.1)	11	22 (4.6)	32 (8)	5 (5.4)	88 (7.8)	13	25 (5.2)	58 (12.5)	5 (2.7)
Skin. tissue and superficial vessel harm[1]	123 (5.9)	4	78 (8.1)	5	59 (12.4)	18 (4.5)	1 (1.1)	45 (4)	4	38 (8)	7 (1.5)	0 (0)
Peri-/post-operative bleeding/ haematoma - did not require re-operation	117 (5.6)	0	29 (3)	3	18 (3.8)	9 (2.3)	2 (2.2)	88 (7.8)	0	61 (12.8)	26 (5.6)	1 (0.5)
Pneumonia	117 (5.6)	4	77 (8)	3	47 (9.9)	25 (6.3)	5 (5.4)	40 (3.6)	5	21 (4.4)	13 (2.8)	6 (3.3)
Falls	87 (4.2)	15	38 (3.9)	19	6 (1.3)	22 (5.5)	10 (10.8)	49 (4.4)	10	17 (3.6)	24 (5.2)	8 (4.4)
Neurological[2]	87 (4.2)	2	46 (4.8)	2.5	33 (6.9)	11 (2.8)	2 (2.2)	41 (3.6)	2	30 (6.3)	9 (1.9)	2 (1.1)
Distended urinary bladder	81 (3.9)	3	52 (5.4)	3	39 (8.2)	12 (3)	1 (1.1)	29 (2.6)	3	24 (5)	5 (1.1)	0 (0)
Pulmonary embolism	64 (3.1)	27	15 (1.5)	12	3 (0.6)	8 (2)	4 (4.3)	49 (4.4)	32	9 (1.9)	15 (3.2)	25 (13.7)
Gastric ulcer	45 (2.2)	6	19 (2)	7	8 (1.7)	10 (2.5)	1 (1.1)	26 (2.3)	5.5	12 (2.5)	13 (2.8)	1 (0.5)
Cardiovascular[3]	40 (1.9)	3	14 (1.4)	13.5	6 (1.3)	5 (1.3)	3 (3.2)	26 (2.3)	3	17 (3.6)	7 (1.5)	2 (1.1)
Pain	36 (1.7)	4	7 (0.7)	3	4 (0.8)	3 (0.8)	0 (0)	29 (2.6)	9	13 (2.7)	13 (2.8)	3 (1.6)
Gastro-intestinal[4]	31 (1.5)	7	9 (0.9)	7	4 (0.8)	4 (1)	1 (1.1)	22 (2)	6.5	11 (2.3)	11 (2.4)	0 (0)
Peri-operative fracture	31 (1.5)	0	8 (0.8)	0	6 (1.3)	2 (0.5)	0 (0)	23 (2)	0	20 (4.2)	3 (0.6)	0 (0)
Renal failure	31 (1.5)	2	20 (2.1)	2	18 (3.8)	2 (0.5)	0 (0)	11 (1)	3	10 (2.1)	1 (0.2)	0 (0)
Deep vein thrombosis	29 (1.4)	42	10 (1)	24.5	0 (0)	6 (1.5)	4 (4.3)	19 (1.7)	46	0 (0)	6 (1.3)	13 (7.1)
Allergic reaction	25 (1.2)	5	8 (0.8)	5	4 (0.8)	4 (1)	0 (0)	17 (1.5)	4	11 (2.3)	4 (0.9)	2 (1.1)
Leg length difference	23 (1.1)	3	4 (0.4)	2.5	3 (0.6)	1 (0.3)	0 (0)	19 (1.7)	3	14 (2.9)	3 (0.6)	2 (1.1)

Gastrointestinal infection	22 (1.1)	9	17 (1.8)	11	8 (1.7)	8 (2)	1 (1.1)	5 (0.4)	4	4 (0.8)	1 (0.2)	0 (0)
Septicaemia	20 (1)	17.5	14 (1.4)	17.5	6 (1.3)	7 (1.8)	1 (1.1)	6 (0.5)	13.5	2 (0.4)	3 (0.6)	1 (0.5)
Mechanical complication[5]	20 (1)	25	7 (0.7)	24	3 (0.6)	4 (1)	0 (0)	13 (1.2)	29	2 (0.4)	5 (1.1)	6 (3.3)
Unclear infection	17 (0.8)	5	11 (1.1)	5	5 (1.1)	6 (1.5)	0 (0)	6 (0.5)	4	5 (1)	1 (0.2)	0 (0)
Respiratory	17 (0.8)	1	9 (0.9)	1	6 (1.3)	3 (0.8)	0 (0)	8 (0.7)	1	7 (1.5)	1 (0.2)	0 (0)
Myocardial infarction	15 (0.7)	7	6 (0.6)	3.5	3 (0.6)	3 (0.8)	0 (0)	9 (0.8)	8	5 (1)	3 (0.6)	1 (0.5)
Mouth and throat infection	12 (0.6)	9	8 (0.8)	9	5 (1.1)	3 (0.8)	0 (0)	4 (0.4)	10	2 (0.4)	2 (0.4)	0 (0)
Peripheral nerve injury[6]	12 (0.6)	2	3 (0.3)	8	1 (0.2)	2 (0.5)	0 (0)	9 (0.8)	2	8 (1.7)	1 (0.2)	0 (0)
Ileus	9 (0.4)	5	4 (0.4)	6	0 (0)	4 (1)	0 (0)	5 (0.4)	4	3 (0.6)	2 (0.4)	0 (0)
Stroke	9 (0.4)	4	4 (0.4)	5	3 (0.6)	1 (0.3)	0 (0)	5 (0.4)	4	3 (0.6)	1 (0.2)	1 (0.5)
Electrolyte imbalance	8 (0.4)	5.5	1 (0.1)	5	1 (0.2)	0 (0)	0 (0)	7 (0.6)	6	2 (0.4)	5 (1.1)	0 (0)
AE caused by anaesthesia[7]	7 (0.3)	1	3 (0.3)	0	3 (0.6)	0 (0)	0 (0)	4 (0.4)	1	3 (0.6)	1 (0.2)	0 (0)
Malnutrition	7 (0.3)	6	4 (0.4)	13	2 (0.4)	1 (0.3)	1 (1.1)	3 (0.3)	6	1 (0.2)	2 (0.4)	0 (0)
Bleeding - not related to surgery[8]	5 (0.2)	11	2 (0.2)	6.5	1 (0.2)	1 (0.3)	0 (0)	3 (0.3)	11	0 (0)	3 (0.6)	0 (0)
Infection other	5 (0.2)	11	4 (0.4)	11	1 (0.2)	3 (0.8)	0 (0)	1 (0.1)	6	0 (0)	1 (0.2)	0 (0)
Bleeding - that required re-operation	5 (0.2)	8	1 (0.1)	8	1 (0.2)	0 (0)	0 (0)	4 (0.4)	5	3 (0.6)	1 (0.2)	0 (0)
Surgical harm – other	3 (0.1)	71	0 (0)	NA	– (–)	– (–)	– (–)	3 (0.3)	71	0 (0)	0 (0)	3 (1.6)
Multi-organ failure	1 (0)	3	1 (0.1)	3	1 (0.2)	0 (0)	0 (0)	0 (0)	NA	– (–)	– (–)	– (–)
Other	10 (0.5)	7.5	3 (0.3)	19	1 (0.2)	1 (0.3)	1 (1.1)	7 (0.6)	7	2 (0.4)	4 (0.9)	1 (0.5)
Total	2093 (100)	8	968 (100)	8	476 (100)	399 (100)	93 (100)	1125 (100)	8	477 (100)	465 (100)	183 (100)

AE, adverse event.

Examples of adverse events in these types; [1] blister, extravasation, phlebitis; [2] acute confusion, hallucination, lethargy; [3] heart failure; [4] obstipation, vomiting, diarrhoea; [5] fracture without a fall, re-operation after several dislocations; [6] foot drop; [7] awareness, aspiration; [8] in connection to urinary catheter or warfarin treatment.
