# Supplementary material for: Timing of adverse events in patients undergoing acute and elective hip arthroplasty surgery: a multicentre cohort study using the Global Trigger Tool
Source: BMJ Open. 2023 Jun 9;13(6):e064794. doi: 10.1136/bmjopen-2022-064794 (PMC10277118; doi:10.1136/bmjopen-2022-064794)
Supplement: Author's manuscript [file bmjopen-2022-064794.draft_revisions.pdf]

BMJ Open

BMJ Open is committed to open peer review. As part of this commitment we make the peer review history of every article we publish publicly available.

When an article is published we post the peer reviewers' comments and the authors' responses online. We also post the versions of the paper that were used during peer review. These are the versions that the peer review comments apply to.

The versions of the paper that follow are the versions that were submitted during the peer review process. They are not the versions of record or the final published versions. They should not be cited or distributed as the published version of this manuscript.

BMJ Open is an open access journal and the full, final, typeset and author-corrected version of record of the manuscript is available on our site with no access controls, subscription charges or pay-per-view fees (http://bmjopen.bmj.com).

If you have any questions on BMJ Open's open peer review process please email info.bmjopen@bmj.com

BMJ Open

Timing of adverse events in patients undergoing acute and elective hip arthroplasty surgery: a multi-centre cohort study

Journal:	*BMJ Open*
Manuscript ID	bmjopen-2022-064794
Article Type:	Original research
Date Submitted by the Author:	16-May-2022
Complete List of Authors:	Magnéli, Martin; Karolinska Institute, Department of Clinical Sciences at Danderyd Hospital; Danderyd University Hospital, Department of Orthopaedics<br>Kelly-Pettersson, Paula ; Karolinska Institutet Department of Clinical Sciences Danderyd Hospital, Orthopaedics; Danderyd University Hospital, Department of orthopaedics<br>Rogmark, Cecilia; Lund University, Department of Clinical Sciences Malmö, Clinical and Molecular Osteoporosis Research Unit; Skåne University Hospital Malmö Orthopedics Clinic, Department of Orthopaedics<br>Gordon, Max; Karolinska Institute, Department of clinical sciences at Danderyd hospital; Danderyd University Hospital, Department of orthopaedics<br>Sköldenberg, Olof; Karolinska Institutet, Department of clinical sciences at Danderyd Hospital; Danderyd University Hospital, Department of orthopaedics<br>Unbeck, Maria; Hogskolan Dalarna; Karolinska Institutet, Department of clinical sciences at Danderyd Hospital
Keywords:	Orthopaedic & trauma surgery < SURGERY, Adult orthopaedics < ORTHOPAEDIC & TRAUMA SURGERY, Hip < ORTHOPAEDIC & TRAUMA SURGERY

SCHOLARONE™ Manuscripts

I, the Submitting Author has the right to grant and does grant on behalf of all authors of the Work (as defined in the below author licence), an exclusive licence and/or a non-exclusive licence for contributions from authors who are: i) UK Crown employees; ii) where BMJ has agreed a CC-BY licence shall apply, and/or iii) in accordance with the terms applicable for US Federal Government officers or employees acting as part of their official duties; on a worldwide, perpetual, irrevocable, royalty-free basis to BMJ Publishing Group Ltd ("BMJ") its licensees and where the relevant Journal is co-owned by BMJ to the co-owners of the Journal, to publish the Work in this journal and any other BMJ products and to exploit all rights, as set out in our licence.

The Submitting Author accepts and understands that any supply made under these terms is made by BMJ to the Submitting Author unless you are acting as an employee on behalf of your employer or a postgraduate student of an affiliated institution which is paying any applicable article publishing charge ("APC") for Open Access articles. Where the Submitting Author wishes to make the Work available on an Open Access basis (and intends to pay the relevant APC), the terms of reuse of such Open Access shall be governed by a Creative Commons licence – details of these licences and which Creative Commons licence will apply to this Work are set out in our licence referred to above.

Other than as permitted in any relevant BMJ Author's Self Archiving Policies, I confirm this Work has not been accepted for publication elsewhere, is not being considered for publication elsewhere and does not duplicate material already published. I confirm all authors consent to publication of this Work and authorise the granting of this licence.

For peer review only

Timing of adverse events in patients undergoing acute and elective hip arthroplasty surgery: a multi-centre cohort study

Martin Magnéli[1,2*], Paula Kelly-Pettersson[1,2*], Cecilia Rogmark[3,4,5], Max Gordon[1,2], Olof Sköldenberg[1,2], Maria Unbeck[1,6]

[1] Karolinska Institutet, Department of Clinical Sciences, Danderyd Hospital, Unit of Orthopaedics, Stockholm, Sweden

[2] Danderyd University Hospital, Department of Orthopaedics, Stockholm, Sweden

[3] Skåne University Hospital, Department of Orthopaedics, Malmö, Sweden

[4] Lund University, Department of Clinical Sciences Malmö, Clinical and Molecular Osteoporosis Research Unit, Malmö, Sweden

[5] Swedish Arthroplasty Register, Centre of Registers Västra Götaland, Gothenburg, Sweden

[6] School of Health and Welfare, Dalarna University, Falun, Sweden

* Contributed equally

Corresponding author:
Martin Magnéli
Karolinska Institutet
Department of Clinical Sciences
Danderyd University Hospital
182 88 Stockholm
Sweden
martin.magneli@ki.se

Word count: 4108

For peer review only - http://bmjopen.bmj.com/site/about/guidelines.xhtml

ABSTRACT

Objective To explore timing in relation to all types of adverse events, severity and preventability for patients undergoing acute and elective hip arthroplasty.

Design A multi-centre cohort study using retrospective record review with Global Trigger Tool methodology in combination with data from several registers.

Setting 24 hospitals in 4 major regions of Sweden.

Participants Patients ≥18 years, undergoing acute or elective total or hemiarthroplasty of the hip were eligible for inclusion. Reviews of weighted samples of 1,998 randomly selected patient records were carried out using Global Trigger Tool methodology. The patients were followed for readmissions up to 90 days post-operatively throughout the whole country.

Results The cohort consisted of 667 acute and 1,331 elective patients. Most AEs occurred peri-and post-operatively (n=2093, 99.1%) and after discharge (n=1142, 54.1%). The median time from the day of surgery to the occurrence of AE was 8 days. The median days for different AE types ranged from 0 to 24.5 for acute and 0 to 71 for elective patients and peaked during different time periods. 40.2% of the AEs, both major and minor, occurred within post-operative day 0 to 5 and 86.9% of the AEs occurred within 30 days. Most of the AEs were deemed to be of major severity (n=1370, 65.5%) or preventable (n=1591, 76%).

Conclusions A wide variability was found regarding the timing of different adverse events with a majority occurring within 30 days. The timing and preventability varied regarding the severity. Most of the adverse events were deemed to be preventable and/or of major severity. To increase patient safety for patients undergoing hip arthroplasty surgery a better understanding of the multi-facetted nature of the timing of AEs in relation to the occurrence of differing AEs is needed.

For peer review only - http://bmjopen.bmj.com/site/about/guidelines.xhtml

Strengths and limitations of this study

- To the best of our knowledge, the current study is among the first to explore timing in relation to all types of adverse events per-, peri- and post-operatively, with associated severity and preventability for patients undergoing acute and elective hip arthroplasty.
- Among the strengths of this study are the multi-centre design with a wide range of patients of all ages undergoing surgery at varying types of hospitals and the 90-day national follow-up irrespective of index hospital.
- Adverse events occurring after 90 days post-operatively or those treated outside the hospital setting were not included.

For peer review only

INTRODUCTION

Hip arthroplasty – "the operation of the century"[1] is an effective surgical treatment of both degenerative hip joint disease and femoral neck fractures. Still, adverse events (AEs) constituting a wide range of conditions can occur during the primary, index hospitalization and even up to years later. Both younger and older patients undergo hip arthroplasty nowadays, but the mean age of around 70 years indicates that a large group of elderly, and potentially frail, patients undergo this surgery. This is particularly true for the group treated due to femoral neck fractures.[2] AEs often entail suffering for the affected individual and the occurrence of preventable AEs reflects the gap between the actual care given and the expected standard of adequate safe care.[3 4]

Timing is an important factor in understanding the occurrence and prevention of AEs. It has been examined in some studies on arthroplasty surgery, but many of these included patients undergoing total primary hip or knee arthroplasty, and elective surgery was most common in these cohorts.[5-13] In contrast Bohl et al.[10] and Malik et al.[14], focused on geriatric patients undergoing hip arthroplasty after acute femoral neck fractures. Many of the timing studies[9-17] have used data from the American College of Surgeons National Surgical Quality Improvement Program with a follow-up period of 30 days post-operatively. This register does not include orthopaedic specific AEs such as dislocations.[9 12] Furthermore, many of these studies have had a rather narrow focus, for example, examining only periprosthetic joint infections[7], pulmonary embolism[8 18], venous thromboembolism[19], periprosthetic femur fractures[20], stroke[17 21], clostridium difficilie colitis[16], acute myocardial infarction[22 23] or a selection of pre-defined AEs.[5 9 10 12 14] In contrast, Parvizi et al.[6] and Yao et al.[11] have examined the occurrence of a broader range of AEs but have not reported the timing for all the AEs that were identified.

Regarding timing of all types of AEs, both surgical and non-surgical, and irrespective of whether they occur pre-, peri- or post-operatively in either acute or elective procedures, we see a lack of knowledge. Furthermore, there is a need to examine timing in relation to the severity and preventability of AEs. Therefore, this study aimed to explore timing in relation to all types of adverse events, severity and preventability for patients undergoing acute and elective hip arthroplasty.

For peer review only - http://bmjopen.bmj.com/site/about/guidelines.xhtml

METHODS

Study design

This sub-study is part of a retrospective multi-centre cohort study.[24] The aim of the main study was to validate the ability of a set of pre-defined ICD-10 codes used on a national level to compare hospitals following primary hip arthroplasties. The method and variables are the same for both the main study and the present study and are briefly described below. A more detailed description is previously published.[24 25]

Participants and setting

The study population consisted of all patients aged ≥18 years undergoing either primary total hip arthroplasty or hemiarthroplasty whether performed electively due to degenerative joint disease or acutely for a femoral neck fracture, that were entered into the SHAR during a three-year period (N=21,774). To increase the probability of selecting primary surgery admissions with the occurrence of at least one AE and to avoid excessive record review on admissions without AEs, we used a weighted sample. In total 20 different selection groups for acute and elective arthroplasties were created.[24]

The study cohort consisted of 2000 patients who had undergone either acute or elective primary surgery. The patients had the index surgery performed in hospitals in one of four major regions in Sweden. Two patients were excluded leaving a final study cohort of 1998 patients. The patients were followed for a maximum of 90 days post-operatively for all in-patient and acute out-patient hospital care throughout the whole country, regardless of the location of the hospital where the index surgery was performed. Therefore, the study cohort had a total of 5423 admissions in 16 of the 21 regions in Sweden, with 69 hospitals involved as individual patients may have had multiple admissions.

Data sources

This study used data from both medical records and three national registers, the National Patient Register, the Swedish Cause of Death Register, and the Swedish Hip Arthroplasty Register (SHAR). In 2019, the SHAR had a completeness of 98% and 97% respectively for total hip

For peer review only - http://bmjopen.bmj.com/site/about/guidelines.xhtml

replacements and hemiarthroplasties.[2] Data on primary surgery, such as date and type of surgery was obtained from the SHAR. This data was then cross-referenced with data from the National Patient Register using the Swedish personal identity number as a unique identifier. With this data, a timeline was created for each patient undergoing primary surgery and was used as a template to identify which admissions were to be reviewed.

We performed a manual retrospective record review using the Swedish version[26] of the Global Trigger Tool, a structured record review methodology.[27] This method is a 2-stage review process.

Definitions

An AE was defined as suffering, physical harm or disease, as well as death related to the index admission and was not an inevitable consequence of the patient´s underlying disease or treatment.

A preventable adverse event was defined as an event that could have been prevented if adequate actions had been taken during the patient's contact with healthcare or social care.[28] A preventable event can be related to acts of commissions or acts of omissions.

The index admission was defined as the orthopaedic admission during which the patient had their primary hip arthroplasty surgery.

The occurrence date of the AE was defined as either the specific date when the sign or symptom first appeared and if this information was not available and the AE occurred after discharge the date when the patient contacted the care giver was then used. The occurrence date could also be the same date as the date of diagnosis.

Retrospective record review process

The record review process is described in detail elsewhere.[25] The recruitment of the ten reviewers (registered nurses, medical students, and physicians) was based on previous experience with record review and/or convenience but all reviewers had with previous experience of orthopaedic care.

A study manual, used as a complement to the Swedish Global Trigger Tool manual[26] with trigger definitions and descriptions, was created to clarify the study-specific interpretations and applications of triggers, definitions, and AE assessments. Triggers are clues or red flags in the medical record that indicating that a potential AE has occurred, for example, readmission within

For peer review only - http://bmjopen.bmj.com/site/about/guidelines.xhtml

30 days, re-operation.

The record review and time frame for the inclusion of an AE covered the period from the start of the admission to a maximum of 90 days post-operatively. During the follow-up period, it was possible to identify AEs in the patient records of patients receiving in-patient and/or out-patient care, irrespective of the speciality, and at any hospital in Sweden.

The reviews were carried out in a 2-stage process using standardized data collection forms, one form for each review stage. In most cases, the same person carried out both stages of the review and the data was entered into a study-specific database.

In the first review stage, all record entries from all healthcare professionals were reviewed. The reviewers screened for the presence of 1 or more of the 38 pre-defined triggers in 5 modules of the Swedish version of the Global Trigger Tool.[26] Only records with triggers indicating at least one potential AE went forward to review stage 2.

In review stage 2, the reviewers sorted the different triggers into potential AEs as more than one of the triggers can be involved in a single AE. Every potential AE was then reviewed separately. To qualify as an AE, a score of three or higher on a 4-point Likert scale was required (1=the AE was not related to index admission, 2=the AE was probably not related to index admission, 3=the AE was probably related to index admission, and 4=the AE was related to index admission). The preventability was also assessed using a similar scale with the same cut-off limit for inclusion regarding preventability.

Several other variables were collected, for example, the timing, type and severity of the AEs. Severity was determined using a slightly modified version of the National Coordinating Council for Medication Error Reporting and Prevention (NCC MERP) Index.[29] NCC MERP Index categories E–I were included as they are related to harm i.e. AE. Events determined to be risks and no-harm incidents, i.e. NCC MERP categories A-D, were excluded.

Patient and public involvement

This study did not involve any patient or public representatives.

Statistical methods

Time from surgery to AE occurrence is presented in median days and interquartile range. The statistical analysis was performed using R 4.1.0 (R Project for Statistical Computing, Vienna,

For peer review only - http://bmjopen.bmj.com/site/about/guidelines.xhtml

Austria), using tidyverse (v. 1.3.1) for dataset manipulation, ggplot2 (v. 3.3.5) for plots and htmlTable (v. 2.2.1) for creating tables.

RESULTS

Demographics

One third of the cohort were patients admitted acutely with a femoral neck fracture and two thirds underwent surgery on an elective basis. Patients with acute admissions were older, received most often hemiarthroplasties and were more often treated at university hospitals (table 1).

Table 1 Demographics

	**All patients n= 1998**	**Acute admitted n= 667**	**Elective admitted n= 1331**
Sex			
Female, n (%)	1250 (62.6)	444 (66.6)	806 (60.6)
Male, n (%)	748 (37.4)	223 (33.4)	525 (39.4)
Age, median years (min–max, IQR)	77 (18–100, 16)	84 (34–100, 10)	73 (18–99, 16)
LOS, median days (min–max, IQR)	6 (1–56, 5)	7 (1–56, 8)	5 (1–52, 3)
Type of surgery			
Total arthroplasty	1435 (71.8)	143 (21.4)	1292 (97.1)
Hemi arthroplasty	563 (28.2)	524 (78.6)	39 (2.9)
Type of hospital, n (%)			
University	630 (31.5)	295 (44.2)	335 (25.2)
Central County Council	556 (27.8)	180 (27.0)	376 (28.2)
County Council	531 (26.6)	109 (16.3)	422 (31.7)
Private*	281 (14.1)	83 (12.4)	198 (14.9)

LOS, length of stay, IQR, interquartile range.
* Most of the surgeries are publicly financed through agreements with the regions.
Note: Weighted samples, the values are not representative for average Swedish orthopaedic care concerning hip arthroplasty.

Adverse event outcomes

In total, 2116 AEs of varying severity were identified in 1171 (58.6%) patients in our weighted sample of which 527 (45.0%) of these patients were affected by more than one AE. The patients admitted acutely sustained 981 (46.4%; min to max, 1–10) AEs and the elective patients 1135 (53.6%; min to max 1–7). The acute patients were affected by an AE to a higher extent compared to elective, 71.4% (476/667) versus 52.3% (696/1331). The patients in the cohort had in total all-cause 3425 re-admissions during the follow-up period. However, some of the re-admissions were not related to the index admission and were therefore not considered to be related to an AE.

For peer review only - http://bmjopen.bmj.com/site/about/guidelines.xhtml

Timing overall and in connection to adverse event types

Four AEs did not have a correct date registered and were excluded, leaving 2112 AEs remaining for the timing analysis, 980 AEs in acute patients and 1132 AEs in elective patients. Nineteen (0.9%) AEs occurred from admission to the day before surgery and 2093 (99.1%) AEs occurred from the day of surgery, including peri-operative AEs, up to 90 days post-operatively.

Of the 2112 AEs, 970 (45.9%) occurred during the index orthopaedic admission, the remaining 1142 (54.1%) AEs occurred after discharge. The AEs occurring after discharged were distributed as follow: 866 (41.0%) within 30 days and 276 (13.1%) between 31 and 90 days after surgery. Of the patients undergoing acute surgery 486 (49.6%) of the 980 AEs occurred during the index admission, compared to elective patients where 484 (42.8%) of 1132 AEs occurred during the index admission.

The median time from day of surgery to an AE was 8 days for the 2093 AEs occurring on the day of surgery and post-operatively. The corresponding median for both acute and elective patients was also 8 days (table 2).

For both acute and elective patients, pressure ulcers, skin, tissue and superficial vessel harm, peri- and post-operative bleeding/haematomas not requiring re-operation and pneumonia were common AEs and peaked during the index admission. Within 30 days after surgery, but after discharge, dislocation of the prothesis and infections such as deep periprosthetic infections, superficial wound infections and urinary tract infections were commonly occurring AEs and peaked in this period for both groups. No AE peaked after 30 days post-operatively for the acute patients, whereas for the elective patients, pulmonary embolisms, deep venous thromboses, mechanical complications and surgical harm – other all peaked after 30 days. Some types of AEs continued to occur at a high rate such as dislocations in both groups after 30 days (table 2).

For peer review only - http://bmjopen.bmj.com/site/about/guidelines.xhtml

Table 2 Overview of identified adverse events for acute respective elective patients during different time frames and time to occurrence, sorted by descending order of types of AEs for all patients

	All		Acute					Elective				
**Type of AEs**	**N (%)**	**Median time from surgery to AE (days)**	**n (%)**	**Median time from surgery to AE (days)**	**Occurred during index admission n (%)**	**Occurred within 30 days but after index n (%)**	**Occurred within 31–90 days n (%)**	**n (%)**	**Median time from surgery to AE (days)**	**Occurred during index admission n (%)**	**Occurred within 30 days but after index n (%)**	**Occurred within 31–90 days n (%)**
Dislocation of the prothesis	274 (13.1)	23	101 (10.4)	23	11 (2.3)	56 (14)	34 (36.6)	173 (15.4)	23	23 (4.8)	83 (17.8)	67 (36.6)
Pressure ulcer	189 (9)	5	143 (14.8)	5	91 (19.1)	46 (11.5)	6 (6.5)	46 (4.1)	4.5	33 (6.9)	11 (2.4)	2 (1.1)
Urinary tract infection	163 (7.8)	8	93 (9.6)	9	39 (8.2)	49 (12.3)	5 (5.4)	70 (6.2)	8	32 (6.7)	36 (7.7)	2 (1.1)
Peri-prosthetic joint infection	149 (7.1)	20	36 (3.7)	19	4 (0.8)	27 (6.8)	5 (5.4)	113 (10)	20	4 (0.8)	80 (17.2)	29 (15.8)
Superficial wound infection	147 (7)	11	59 (6.1)	11	22 (4.6)	32 (8)	5 (5.4)	88 (7.8)	13	25 (5.2)	58 (12.5)	5 (2.7)
Skin. tissue and superficial vessel harm[1]	123 (5.9)	4	78 (8.1)	5	59 (12.4)	18 (4.5)	1 (1.1)	45 (4)	4	38 (8)	7 (1.5)	0 (0)
Peri-/post-operative bleeding/ haematoma - did not require re-operation	117 (5.6)	0	29 (3)	3	18 (3.8)	9 (2.3)	2 (2.2)	88 (7.8)	0	61 (12.8)	26 (5.6)	1 (0.5)
Pneumonia	117 (5.6)	4	77 (8)	3	47 (9.9)	25 (6.3)	5 (5.4)	40 (3.6)	5	21 (4.4)	13 (2.8)	6 (3.3)
Falls	87 (4.2)	15	38 (3.9)	19	6 (1.3)	22 (5.5)	10 (10.8)	49 (4.4)	10	17 (3.6)	24 (5.2)	8 (4.4)
Neurological[2]	87 (4.2)	2	46 (4.8)	2.5	33 (6.9)	11 (2.8)	2 (2.2)	41 (3.6)	2	30 (6.3)	9 (1.9)	2 (1.1)
Distended urinary bladder	81 (3.9)	3	52 (5.4)	3	39 (8.2)	12 (3)	1 (1.1)	29 (2.6)	3	24 (5)	5 (1.1)	0 (0)
Pulmonary embolism	64 (3.1)	27	15 (1.5)	12	3 (0.6)	8 (2)	4 (4.3)	49 (4.4)	32	9 (1.9)	15 (3.2)	25 (13.7)
Gastric ulcer	45 (2.2)	6	19 (2)	7	8 (1.7)	10 (2.5)	1 (1.1)	26 (2.3)	5.5	12 (2.5)	13 (2.8)	1 (0.5)
Cardiovascular[3]	40 (1.9)	3	14 (1.4)	13.5	6 (1.3)	5 (1.3)	3 (3.2)	26 (2.3)	3	17 (3.6)	7 (1.5)	2 (1.1)
Pain	36 (1.7)	4	7 (0.7)	3	4 (0.8)	3 (0.8)	0 (0)	29 (2.6)	9	13 (2.7)	13 (2.8)	3 (1.6)
Gastro-intestinal[4]	31 (1.5)	7	9 (0.9)	7	4 (0.8)	4 (1)	1 (1.1)	22 (2)	6.5	11 (2.3)	11 (2.4)	0 (0)
Peri-operative fracture	31 (1.5)	0	8 (0.8)	0	6 (1.3)	2 (0.5)	0 (0)	23 (2)	0	20 (4.2)	3 (0.6)	0 (0)
Renal failure	31 (1.5)	2	20 (2.1)	2	18 (3.8)	2 (0.5)	0 (0)	11 (1)	3	10 (2.1)	1 (0.2)	0 (0)
Deep vein thrombosis	29 (1.4)	42	10 (1)	24.5	0 (0)	6 (1.5)	4 (4.3)	19 (1.7)	46	0 (0)	6 (1.3)	13 (7.1)
Allergic reaction	25 (1.2)	5	8 (0.8)	5	4 (0.8)	4 (1)	0 (0)	17 (1.5)	4	11 (2.3)	4 (0.9)	2 (1.1)
Leg length difference	23 (1.1)	3	4 (0.4)	2.5	3 (0.6)	1 (0.3)	0 (0)	19 (1.7)	3	14 (2.9)	3 (0.6)	2 (1.1)
Gastrointestinal infection	22 (1.1)	9	17 (1.8)	11	8 (1.7)	8 (2)	1 (1.1)	5 (0.4)	4	4 (0.8)	1 (0.2)	0 (0)
Septicaemia	20 (1)	17.5	14 (1.4)	17.5	6 (1.3)	7 (1.8)	1 (1.1)	6 (0.5)	13.5	2 (0.4)	3 (0.6)	1 (0.5)

For peer review only

Mechanical complication[5]	20 (1)	25	7 (0.7)	24	3 (0.6)	4 (1)	0 (0)	13 (1.2)	29	2 (0.4)	5 (1.1)	6 (3.3)
Unclear infection	17 (0.8)	5	11 (1.1)	5	5 (1.1)	6 (1.5)	0 (0)	6 (0.5)	4	5 (1)	1 (0.2)	0 (0)
Respiratory	17 (0.8)	1	9 (0.9)	1	6 (1.3)	3 (0.8)	0 (0)	8 (0.7)	1	7 (1.5)	1 (0.2)	0 (0)
Myocardial infarction	15 (0.7)	7	6 (0.6)	3.5	3 (0.6)	3 (0.8)	0 (0)	9 (0.8)	8	5 (1)	3 (0.6)	1 (0.5)
Mouth and throat infection	12 (0.6)	9	8 (0.8)	9	5 (1.1)	3 (0.8)	0 (0)	4 (0.4)	10	2 (0.4)	2 (0.4)	0 (0)
Peripheral nerve injury[6]	12 (0.6)	2	3 (0.3)	8	1 (0.2)	2 (0.5)	0 (0)	9 (0.8)	2	8 (1.7)	1 (0.2)	0 (0)
Ileus	9 (0.4)	5	4 (0.4)	6	0 (0)	4 (1)	0 (0)	5 (0.4)	4	3 (0.6)	2 (0.4)	0 (0)
Stroke	9 (0.4)	4	4 (0.4)	5	3 (0.6)	1 (0.3)	0 (0)	5 (0.4)	4	3 (0.6)	1 (0.2)	1 (0.5)
Electrolyte imbalance	8 (0.4)	5.5	1 (0.1)	5	1 (0.2)	0 (0)	0 (0)	7 (0.6)	6	2 (0.4)	5 (1.1)	0 (0)
AE caused by anaesthesia[7]	7 (0.3)	1	3 (0.3)	0	3 (0.6)	0 (0)	0 (0)	4 (0.4)	1	3 (0.6)	1 (0.2)	0 (0)
Malnutrition	7 (0.3)	6	4 (0.4)	13	2 (0.4)	1 (0.3)	1 (1.1)	3 (0.3)	6	1 (0.2)	2 (0.4)	0 (0)
Bleeding - not related to surgery[8]	5 (0.2)	11	2 (0.2)	6.5	1 (0.2)	1 (0.3)	0 (0)	3 (0.3)	11	0 (0)	3 (0.6)	0 (0)
Infection other	5 (0.2)	11	4 (0.4)	11	1 (0.2)	3 (0.8)	0 (0)	1 (0.1)	6	0 (0)	1 (0.2)	0 (0)
Bleeding - that required re-operation	5 (0.2)	8	1 (0.1)	8	1 (0.2)	0 (0)	0 (0)	4 (0.4)	5	3 (0.6)	1 (0.2)	0 (0)
Surgical harm – other	3 (0.1)	71	0 (0)	NA	– (–)	– (–)	– (–)	3 (0.3)	71	0 (0)	0 (0)	3 (1.6)
Multi-organ failure	1 (0)	3	1 (0.1)	3	1 (0.2)	0 (0)	0 (0)	0 (0)	NA	– (–)	– (–)	– (–)
Other	10 (0.5)	7.5	3 (0.3)	19	1 (0.2)	1 (0.3)	1 (1.1)	7 (0.6)	7	2 (0.4)	4 (0.9)	1 (0.5)
Total	2093 (100)	8	968 (100)	8	476 (100)	399 (100)	93 (100)	1125 (100)	8	477 (100)	465 (100)	183 (100)

AE, adverse event.

Examples of adverse events in these types; [1] blister, extravasation, phlebitis; [2] acute confusion, hallucination, lethargy; [3] heart failure; [4] obstipation, vomiting, diarrhoea; [5] fracture without a fall, re-operation after several dislocations; [6] foot drop; [7] awareness, aspiration; [8] in connection to urinary catheter or warfarin treatment.

For peer review only

For peer review only - http://bmjopen.bmj.com/site/about/guidelines.xhtml

The 8 most common types of AEs and their median day of occurrence are shown in figure 1. Of these, peri-/post-operative bleeding/haematoma had the earliest day of occurrence at 3 (interquartile range, 12) days for acute patients and 0 (7) days for elective patients. The second earliest occurring AE was skin, tissue and superficial vessel harm with a median of 5 (9) for acute and 4 (4) days for the elective patients. Dislocations had the longest median time to occurrence with 23 (24 versus 29) days for both groups.

Add figure 1 here.

Timing outcomes within 5 days post-operatively

In total, 40.2% of both minor and major AEs (n=842/2093) occurred either on the day of surgery or during the following 5 post-operative days (table 3). Some types of AEs were only common early in the 5-day time frame, for example, peri-operative haemorrhage, peri-operative fractures and dislocation on post-operative day 1 while some other AEs such as skin, tissue and superficial vessel harm and distended urinary bladder were frequent from the day of surgery and onwards. Later in the time frame, infections such as pneumonia, urinary tract infections and superficial wound infections began to commonly occur.

Table 3 Peri-operative and early post-operative adverse events (AEs), the 5 most common AE types per day

Type of AE, n	POD 0	POD 1	POD 2	POD 3	POD 4	POD 5
Peri-operative haemorrhage	59					
Peri-operative fracture	25					
Skin, tissue and superficial vessel harm	11	14		17	10	11
Neurological	9	20	18			
Distended urinary bladder	8	9	16	17	8	
Dislocation		14				
Pressure ulcer		14	21	30	17	15
Pneumonia			27	18		7
Urinary tract infection			14	11	13	5
Superficial wound infection					12	5
Total for the 5 most common per day, (%) of total per day	112 (64.0)	71 (48.6)	96 (56.8)	93 (58.5)	60 (55.0)	43 (51.2)
Total for all AEs per day	175	146	169	159	109	84

POD, post-operative day.

Timing and preventability in relation to severity

Preventability and timing varied within and between the acute and elective groups, as well as, for the different AE types regarding severity. Most of the AEs were deemed to be of major severity (n=1370, 65.5%, NCC MERP categories F-I) or preventable (n=1591, 76.0%). The

For peer review only - http://bmjopen.bmj.com/site/about/guidelines.xhtml

proportion of major AEs was higher among the elective patients compared to the acute, 72.9% versus 56.8%. On the contrary, the proportion of AEs that contributed to death was higher among the acute patients, 3.9% versus 0.5%. All deaths in the elective group were deemed preventable and the AEs contributing to death began to occur later (median days 20.5) in contrast to the 65.9% preventable AEs for the acute group with a median of 15 days (table 4). Median days to death was in total 29 days for 28 acute patients and 25 days for 4 elective patients. Examples of AEs resulting in permanent harm to some degree, classified according to the Swedish patient insurance assessment standards, were dislocations, deep peri-prosthetic infections, fractures, thromboses/embolisms, and leg length difference.

In total, a third of the AEs were classified as minor (NCC MERP category E) including, for example, pressure ulcers, urinary tract infections, skin harm, falls with minor injury, distended urinary bladders, and intravenous infiltrations. In total, minor AEs and AEs that required intervention necessary to sustain life within 60 minutes (NCC MERP category H) occurred earlier (median 4 respective 1 day) compared to the other NCC MERP categories. Among the acute patients, minor AEs occurred more often but somewhat later and were assessed to be more preventable compared those in elective patients (table 4).

Table 4 Number, proportion, and timing of adverse events (AEs) and preventable AEs in relation to severity

	Severity according to NCC MERP					
Groups	E	F	G	H	I	Total
**Adverse events, all patients**						
N (%) of AEs	723 (34.5)	794 (37.9)	518 (24.7)	14 (0.7)	44 (2.1)	2093 (100)
Median (IQR) days	4 (7)	8 (15)	21 (27)	1 (3)	15.5 (18.75)	8 (16)
N (%) of preventable AEs	528 (73.0)	541 (68.1)	482 (93.0)	11 (78.6)	29 (65.9)	1,591 (76.0)
Median (IQR), days, preventable AEs	4 (7)	8 (14)	21 (27)	1 (2.5)	16 (18)	9 (17)
**Adverse events, acute patients**						
N (%) of AEs	418 (43.2)	335 (34.6)	169 (17.5)	8 (0.8)	38 (3.9)	968 (100)
Median (IQR) days	5 (8)	7 (13)	22 (23)	1 (1.75)	15 (20.25)	8 (14)
N (%) of preventable AEs	321 (76.8)	243 (72.5)	155 (91.7)	8 (100)	23 (60.5)	750 (77.5)
Median (IQR), days, preventable AEs	5 (7)	7 (12.5)	22 (20.5)	1 (1.75)	15 (19.5)	8 (14.75)
**Adverse events, elective patients**						
N (%) of AEs	305 (27.1)	459 (40.8)	349 (31.0)	6 (0.5)	6 (0.5)	1125 (100)
Median (IQR) days	3 (6)	8 (16)	21 (31)	1.5 (6.25)	20.5 (13)	8 (18)
N (%) of preventable AEs	207 (67.9)	298 (64.9)	327 (93.7)	3 (50.0)	6 (100)	841 (74.8)
Median (IQR), days, preventable AEs	3 (5)	8.5 (15.75)	21 (29)	1 (14.5)	20.5 (13)	10 (19)

NCC MERP, National Coordinating Council for Medication Error Reporting and Prevention Index.
E, Contributed to or resulted in temporary harm; F, Contributed to or resulted in temporary harm that prolonged hospitalization, required out-patient care or readmission; G, Contributed to or resulted in permanent patient harm; H, Required intervention necessary to sustain life within 60 minutes; I, Contributed or resulted in the patient's death
IQR, interquartile range.

For peer review only - http://bmjopen.bmj.com/site/about/guidelines.xhtml

DISCUSSION

To the best of our knowledge, this is the first study to explore all types of AEs with associated timing data, both in total and for acute and elective patients. Most of the AEs occurred after the index admission for both groups. Eighty-seven percent of the AEs occurred within 30 days post-operatively. The overall median time of 8 days from the day of surgery to an AE was the same for both acute and elective patients. Pressure ulcers and skin, tissue and superficial vessel harm were common and peaked for both acute and elective patients during the index admission. Dislocations and deep periprosthetic joint infections were common reasons for readmissions within 30 days after surgery and peaked during this time frame. Dislocations continued to occur to a high extent after 30 days. Forty percent of all AEs, both major and minor, occurred from the day of surgery and up to and including the fifth post-operative day. The timing and preventability varied regarding the severity. Most of the AEs were deemed to be preventable and/or of major severity.

Previous timing studies examining patients undergoing arthroplasty have used varying data collection methods, included different types of arthroplasty surgery, often with a focus on total hip replacements in elective patients, and the criteria for AEs, timing and follow-up periods differ. Furthermore, some studies do not report data for hip arthroplasty separately. This makes comparison of the outcomes somewhat difficult.

Type of adverse events and timing in general

A wide variability was found regarding the timing of AEs. Peri-/post-operative bleeding/ haematoma, peri-operative fracture, AEs caused by anaesthesia, respiratory AEs, renal failure, peripheral nerve injuries and neurological AEs occurred already within 2 days post-operatively. The latest occurring AEs included surgical harms of other types, deep vein thromboses, mechanical complications, dislocations and peri-prosthetic infections which ranged from median 20 to 71 days. Some AEs continued to occur up until the end of the follow-up period.

Dislocation was the most common AE for elective patients and the second most common for acute patients. It occurred at a median time of 3 weeks and in 9 of 10 cases after discharge. This AE is seldom reported in timing studies, for example, the commonly used National Surgical Quality Improvement Program does not include dislocations and other orthopaedic-specific AEs.[9 12] However, Ali et al.[30] also found that dislocation was one of the most common AEs which clustered early after discharge. In their study, after 30 days post-operatively 74% of the

For peer review only - http://bmjopen.bmj.com/site/about/guidelines.xhtml

dislocations requiring readmission had occurred and this was higher than the 51% in the present study. Even after 30 days, dislocations continued to occur to a high extent.

Other common AEs were infections such as periprosthetic, urinary tract and superficial wound infections and the majority occurred after discharge. The median time to superficial wound infections and periprosthetic infections were 11 versus 20 days and the corresponding for Malik et al.[14] were 16 versus 23 days. Bohl et al.[9 10] reported in two studies surgical site infections with median days of 17 and 16, respectively. Our median time to urinary tract infections is in line with results from other studies with range of 7 to 8 days.[9 10 14]

Contrary to other AEs, pulmonary embolism and deep vein thrombosis occurred relatively late at 4 and 6 weeks respectively. The longer time to the occurrence of deep vein thrombosis compared to pulmonary embolism can also be seen in other studies.[5 9 10 14] In contrast, these two AE types occurred later in our cohort than in other studies where median days to pulmonary embolism and deep vein thrombosis ranged from 3-5 days and 6-9 days, respectively.[5 9 10 14] A partial explanation can be that we had a longer follow-up period which affects the median day outcomes.

Several of our most common AE types, such as, pressure ulcers, skin, tissue and superficial vessel harm, falls, neurological harm, and distended urinary bladder are not covered by similar timing studies on AEs in hip arthroplasty patients. These AEs, which mostly peaked during the index admission, were often classified as minor and preventable but not directly related to the surgical intervention. Nevertheless, also minor AEs may lead to suffering for the patient. To reduce the number of AEs occurring in orthopaedic care, it is important to analyse AEs also from a timing perspective and work proactively pre-, peri- and post-operatively in interprofessional teams both inside and outside the operating room.[31]

Two timing studies focused on geriatric patients with hip fractures in general[14] and treated with arthroplasty.[10] Although, timing of specific AEs was quite similar, pulmonary embolism, deep vein thrombosis and sepsis occurred somewhat later and superficial wound infections occurred somewhat earlier in our acute cohort. The patterns of AE types in relation to early or late onset were mostly similar.

Timing during and after index admission, early and late onset

We found that less than half of all AEs occurred during the index admission. The median length of index stay is longer in our study compared to several others. This can be explained by the

For peer review only - http://bmjopen.bmj.com/site/about/guidelines.xhtml

fact that we included acute patients and admissions with longer length of stay were over-represented due to the sampling technique used in the main study. Nevertheless, other studies with shorter length of stay found that most of the AEs occurred during the index admission.[9 10 12] In contrast, Yao et al.[11] had a higher AE occurrence after discharge when studying a cohort of home-admitted patients. As we are moving toward shorter hospital stays and an increased amount of day-surgery, the risk of major AEs occurring outside the hospitals increases. Therefore, a careful follow-up plan after discharge can be of value. Co-production of healthcare outcomes by the healthcare professionals and the patient as an important participating partner may be a significant intervention in the preventive work to reduce the occurrence of AEs and increase patient safety.[32] The patients, and in some cases their significant others and the staff at nursing homes, need to be well informed regarding which signs and symptoms of AEs to be aware of and how to manage self-care which are central aspects during the rehabilitation period.

Most of the AEs in the present study occurred within 30 days post-operatively. Many other studies had a follow-up period of only 30 days and most of the AEs would probably be detected during this period. However, this time frame may lead to a risk of underestimation of the incidence of AEs and a bias towards the earlier-diagnosed AEs as some AE types, for example, surgical site infections did not plateau within this specific follow-up period.[9 10 14]

Nearly half of all AEs occurred within the first 5 days post-operatively. Still, none of the 4 "catastrophic" AEs, pre-defined by Johnson et al.[12] namely pulmonary embolism, myocardial infarction or cardiac arrest, cerebrovascular accident, or death were among our 5 most common AEs during this time frame. The corresponding results for Parvizi et al.[6] was 93% for life-threating medical AEs. Belmont et al.´s[13] findings showed that 77% of total hip arthroplasty patients who had an adverse cardiac event in the form of myocardial infarction or cardiac arrest experienced it within 3 days after surgery.

Severity and preventability

The timing and preventability varied regarding the severity for the acute and elective patients. We assessed severity with a widely used scale in AE studies using the Global Trigger Tool record review methodology.[27] The severity of each AE was assessed independently, and a specific type of AE, for example pressure ulcers, falls and urinary tract infections could be assessed as having varying degrees of severity depending on the harm caused. Parvizi et al.[6] and Yao et al.[11] classified all, for example, pneumonia as minor AEs while we classified them

For peer review only - http://bmjopen.bmj.com/site/about/guidelines.xhtml

as major. On the other hand, Parvizi et al.[6] defined AEs that resulted in, for example, prolonged hospital stays as minor while we classified these AEs as major (NCC MERP F).

In contrast to other studies[5 6 11-14], we did not include death as a separate AE type as we considered death to be the consequence of an AE and belongs to the severity classification. The median time to death was around 4 weeks for both groups. For some patients more than one AE may have contributed to their death (NCC MERP category I).

Further research is needed to explore the multi-facetted nature of the timing in relation to the occurrence of minor and major AEs, both surgical and non-surgical, during the pre-, peri- or post-operative periods of patient care. A multi-pronged approach is required in the prevention of AEs[14], as most AEs were deemed to be preventable, and patients were commonly affected by more than one AE. To increase patient safety for this patient group additional research is needed regarding the implementation and evaluation of timely targeted interventions during the varying phases of the patient-care process, focusing on preventable AEs.

Limitations and strengths

The study has several limitations. We have not included AEs occurring after 90 days post-operatively. Using record review as the data collection method may have led to an underestimation of AEs due to underreporting. Furthermore, AEs detected and treated outside the hospital setting may have been missed. Probably, these are minor AEs as severe AEs are more likely to be treated in a hospital setting. Even though we reported the time to occurrence in most cases, some AEs occurring after discharge may have had a delayed timing date if the patient was not specific enough when the first symptom of the AE occurred. In these cases, the date of when the patient first contacted the care giver was used.

The multi-centre design with a wide range of patients of all ages and types of hospitals can be considered as a strength. The 90-day national follow-up regardless of index hospital should be sufficient to detect most acute, sub-acute and rare AEs. To ensure the reliability and validity of our data, the study has been closely monitored to check the correctness and completeness of the data, enabling for a good control of the review process. We used a stringent definition of what constitutes an AE and attained very good kappa values in the review process[24]. We only included AEs related to the care given and excluded conditions judged to be related to an underlying disease or condition unless the disease or condition deteriorated due to healthcare.

For peer review only - http://bmjopen.bmj.com/site/about/guidelines.xhtml

We believe our results to be valid and generalizable at least to Western world patients and/or publicly financed healthcare.

For peer review only

For peer review only - http://bmjopen.bmj.com/site/about/guidelines.xhtml

Acknowledgements

The authors wish to thank Marie Ax, Susanne Hansson, Zara Hedlund, Mirta Stupin, Tim Hansson, Lovisa Hult-Ericson, and Christina Jansson for their help and support with the review records. We would also like to thank all departmental managers for providing access to the patient records and Per Nydert for his help with the study database.

Contributors

MM collected data, analysed the data, and critically revised the manuscript.

PKP interpreted the data and contributed to the drafting of the work.

CR contributed to the design of the study and contributed to the drafting of the work.

MG contributed to the design of the study, collected data, and critically revised the manuscript.

OS contributed to the design of the study, collected data, and critically revised the manuscript.

MU contributed to the design of the study, monitored the reviews, analysed and interpreted the data, and was mainly responsible for the drafting of the work.

All authors have approved the final version of the manuscript and agree to be accountable for all aspects of the work.

Competing interests

None to declare.

Data sharing statement

No additional data is available.

Ethical approval

Ethical approval was provided by the Regional Ethics Committee of Gothenburg (516-13 and T732-13). Permission for data access for the reviewers was granted by the head of each respective unit. The patients did not provide an informed consent to the record review.

For peer review only

Funding

This work was supported by the Swedish Patient Insurance. The funder has not been involved in any part of the study, in either writing the manuscript or the decision to submit the manuscript for publication.

Patient and Public involvement

Patients and/or the public were not involved in the design, conduct, reporting or dissemination plans of our research.

Patient consent for publication

Not applicable.

ORCHID IDs

Martin Magnéli https://orchid.org/ 0000-0003-0341-0227

Paula Kelly-Pettersson https://orchid.org/0000-0003-3685-2953

Cecilia Rogmark https://orchid.org/0000-0002-6556-4320

Max Gordon https://orchid.org/ 0000-0002-8080-5815

Olof Sköldenberg https://orchid.org/ 0000-0003-4155-8425

Maria Unbeck https://orchid.org/ 0000-0002-5090-0352

For peer review only

REFERENCES

1 Learmonth ID, Young C, Rorabeck C. The operation of the century: total hip replacement. *Lancet* 2007;370:1508-19.

2 W-Dahl A, Kärrholm J, Nauclér E, *et al*. Annual report 2021. The Swedish Arthroplasty Register. Available: https://registercentrum.blob.core.windows.net/slr/r/SAR-Annual-Report-2021-SJlAFmlRl5.pdf [Accessed 10 May 2022].

3 Duarte Sda C, Stipp MA, da Silva MM, *et al*. Adverse events and safety in nursing care. *Rev Bras Enferm* 2015;68:136-46, 144-54.

4 Pettersson PK, Skoldenberg O, Samuelsson B, *et al*. The identification of adverse events in hip fracture patients using the Global Trigger Tool: A prospective observational cohort study. *Int J Orthop Trauma Nurs* 2020;38:100779.

5 Mantilla CB, Horlocker TT, Schroeder DR, *et al*. Frequency of myocardial infarction, pulmonary embolism, deep venous thrombosis, and death following primary hip or knee arthroplasty. *Anesthesiology* 2002;96:1140-6.

6 Parvizi J, Mui A, Purtill JJ, *et al*. Total joint arthroplasty: When do fatal or near-fatal complications occur? *J Bone Joint Surg Am* 2007;89a:27-32.

7 Pulido L, Ghanem E, Joshi A, *et al*. Periprosthetic joint infection: The incidence, timing, and predisposing factors. *Clin Ortho Relat Res* 2008;466:1710-15.

8 Parvizi J, Huang R, Raphael IJ, et al. Timing of symptomatic pulmonary embolism with warfarin following arthroplasty. *J Arthroplasty* 2015;30:1050-3.

9 Bohl DD, Ondeck NT, Basques BA, *et al*. What is the timing of general health adverse events that occur after total joint arthroplasty? *Clin Orthop Relat Res* 2017;475:2952-59.

10 Bohl DD, Samuel AM, Webb ML, *et al*. Timing of adverse events following geriatric hip fracture surgery: A Study of 19,873 patients in the American college of surgeons national surgical quality improvement program. *Am J Orthop (Belle Mead NJ)* 2018;47.

11 Yao DH, Keswani A, Shah CK, *et al*. Home discharge after primary elective total joint arthroplasty: postdischarge complication timing and risk factor analysis. *J Arthroplasty* 2017;32:375-80.

12 Johnson DJ, Hartwell MJ, Weiner JA, *et al*. Which postoperative day after total joint arthroplasty are catastrophic events most likely to occur? *J Arthroplasty* 2019;34:2466-72.

13 Belmont PJ, Goodman GP, Kusnezov NA, *et al*. Postoperative myocardial infarction and cardiac arrest following primary total knee and hip arthroplasty: rates, risk factors, and time of occurrence. *J Bone Joint Surg Am* 2014;96a:2025-31.

For peer review only - http://bmjopen.bmj.com/site/about/guidelines.xhtml

14 Malik AT, Quatman CE, Phieffer LS, *et al*. Timing of complications following surgery for geriatric hip fractures. *J Clin Orthop Trauma* 2019;10:904-11.

15 Minhas SV, Kester BS, Lovecchio FC, et al. Nationwide 30-day readmissions after elective orthopedic surgery: reasons and implications. *J Healthc Qual* 2017;39:34-42.

16 Bovonratwet P, Bohl DD, Malpani R, *et al*. Incidence, risk factors, and impact of clostridium difficile colitis following primary total hip and knee arthroplasty. *J Arthroplasty* 2018;33:205-10.e1.

17 Haynes MS, Alder KD, Bellamkonda K, *et al*. Incidence, predictors, and timing of post-operative stroke following elective total hip arthroplasty and total knee arthroplasty. *PLoS One* 2020;15:e0239239.

18 Hu C, Liu C, Wang Y, *et al*. The timing of symptomatic pulmonary embolism in patients with nonwarfarin anticoagulants following elective primary total joint arthroplasty. *J Arthroplasty* 2020;35:1703-07.

19 Shohat N, Goel R, Ludwick L, *et al*. Time to venous thromboembolism events following total hip arthroplasty: A comparison between aspirin and warfarin. *J Arthroplasty* 2022. [Published Online First: 2022/02/13]

20 Lygrisse KA, Gaukhman GD, Teo G, et al. Is surgical approach for primary total hip arthroplasty associated with timing, incidence, and characteristics of periprosthetic femur fractures? *J Arthroplasty* 2021;36:3305-11.

21 Lalmohamed A, Vestergaard P, Cooper C, *et al*. Timing of stroke in patients undergoing total hip replacement and matched controls: a nationwide cohort study. *Stroke* 2012;43:3225-9.

22 Lalmohamed A, Vestergaard P, Klop C, *et al*. Timing of acute myocardial infarction in patients undergoing total hip or knee replacement: a nationwide cohort study. *Arch Intern Med* 2012;172:1229-35.

23 Gandhi R, Petruccelli D, Devereaux PJ, et al. Incidence and timing of myocardial infarction after total joint arthroplasty. *J Arthroplasty* 2006;21:874-7.

24 Magnéli M, Unbeck M, Rogmark C, et al. Validation of adverse events after hip arthroplasty: a Swedish multi-centre cohort study. *BMJ Open* 2019;9:e023773.

25 Hommel A, Magneli M, Samuelsson B, et al. Exploring the incidence and nature of nursing-sensitive orthopaedic adverse events: A multicenter cohort study using Global Trigger Tool. *Int J Nurs Stud* 2020;102:103473.

For peer review only - http://bmjopen.bmj.com/site/about/guidelines.xhtml

26 SALAR. *Marker-based record review: to identify and measure harm in healthcare* [in Swedish: Markörbaserad journalgranskning: för att identifiera och mäta skador i vården]. Stockholm: Swedish Association of Local Authorities and Regions (SALAR), 2012.

27 Griffin FA, Resar, R.K. *IHI Global Trigger Tool for measuring adverse events* (second edition). IHI Innovation Series white paper. Cambridge, MA: Institute for Healthcare Improvement, 2009.

28 Swedish Codes of Statutes. Patient Safety Act (2010:659) [In Swedish: Patientsäkerhetslag 2010:659]. Available: https://www.riksdagen.se/sv/dokument-lagar/dokument/svensk-forfattningssamling/patientsakerhetslag-2010659_sfs-2010-659 [Accessed 10 May 2022].

29 National Coordinating Council for Medication Error Reporting and Prevention. Available: https://www.nccmerp.org/types-medication-errors [Accessed 10 May 2022].

30 Ali AM, Loeffler MD, Aylin P, *et al*. Timing of readmissions after elective total hip and knee arthroplasty: Does a 30-day all-cause rate capture surgically relevant readmissions? *J Arthroplasty* 2021;36:728-33.

31 Unbeck M, Dalen N, Muren O, *et al*. Healthcare processes must be improved to reduce the occurrence of orthopaedic adverse events. *Scand J Caring Sci* 2010;24:671-7.

32 World Health Organization. Exploring patient participartion in reducing health-care-related safety risks. 2013. Available: https://www.euro.who.int/__data/assets/pdf_file/0010/185779/e96814.pdf [Accessed 29 April 2022].

For peer review only

Legends

Figure 1. Median days from the day of surgery for the 8 most common types of adverse events up to 90 days post-operatively.

For peer review only

For peer review only - http://bmjopen.bmj.com/site/about/guidelines.xhtml

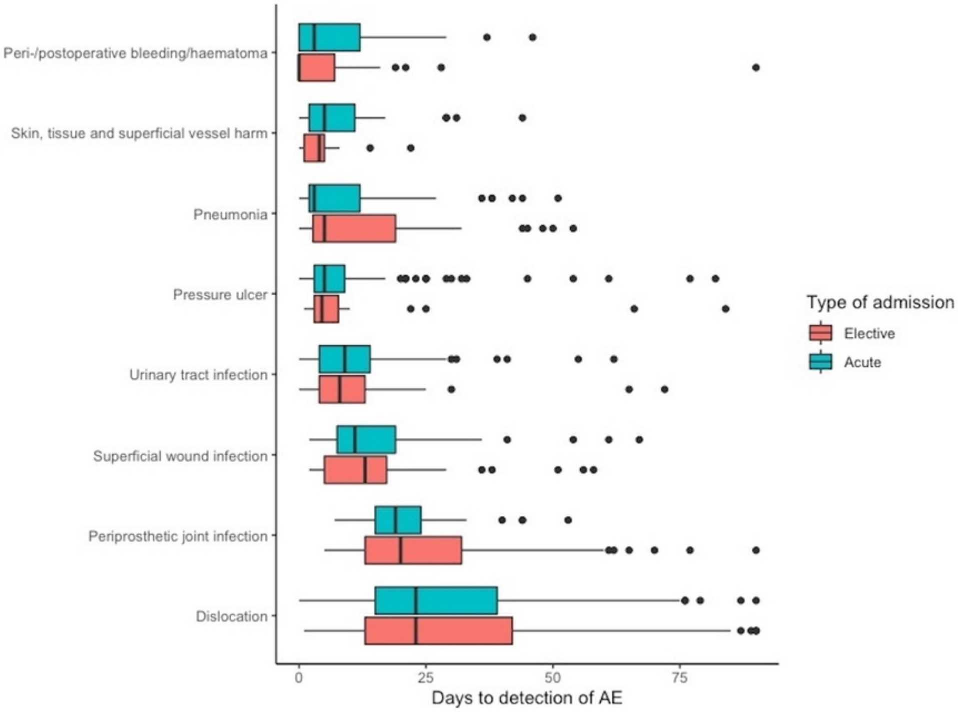

Figure 1 Median days from the day of surgery for the 8 most common types of adverse events up to 90 days post-operatively. The median (interquartile) day of occurrence for acute respective elective patients was for peri- or post-operative bleeding/haematoma 3 (7), 0 (12), skin, tissue and superficial vessel harm 5 (9), 4 (4), pneumonia 3 (10), 5 (16.25), pressure ulcer 5 (6), 4.5 (4.75), urinary tract infection 9 (10), 8 (9), superficial wound infection 11 (11.5), 12 (11.75), peri-prosthetic joint infection 19 (9), 20 (19), and dislocation 23 (24), 23 (29).

76x57mm (300 x 300 DPI)

STROBE Statement—Checklist of items that should be included in reports of ***cohort studies***

	Item No	Recommendation	Page No
**Title and abstract**	1	(*a*) Indicate the study's design with a commonly used term in the title or the abstract	1
		(*b*) Provide in the abstract an informative and balanced summary of what was done and what was found	2
**Introduction**			
Background/rationale	2	Explain the scientific background and rationale for the investigation being reported	4
Objectives	3	State specific objectives, including any prespecified hypotheses	4
**Methods**			
Study design	4	Present key elements of study design early in the paper	5
Setting	5	Describe the setting, locations, and relevant dates, including periods of recruitment, exposure, follow-up, and data collection	5
Participants	6	(*a*) Give the eligibility criteria, and the sources and methods of selection of participants. Describe methods of follow-up<br>(*b*) For matched studies, give matching criteria and number of exposed and unexposed	5
Variables	7	Clearly define all outcomes, exposures, predictors, potential confounders, and effect modifiers. Give diagnostic criteria, if applicable	6
Data sources/ measurement	8*	For each variable of interest, give sources of data and details of methods of assessment (measurement). Describe comparability of assessment methods if there is more than one group	6-7
Bias	9	Describe any efforts to address potential sources of bias	NA
Study size	10	Explain how the study size was arrived at	5 (references)
Quantitative variables	11	Explain how quantitative variables were handled in the analyses. If applicable, describe which groupings were chosen and why	NA
Statistical methods	12	(*a*) Describe all statistical methods, including those used to control for confounding<br>(*b*) Describe any methods used to examine subgroups and interactions<br>(*c*) Explain how missing data were addressed<br>(*d*) If applicable, explain how loss to follow-up was addressed<br>(*e*) Describe any sensitivity analyses	7
**Results**			
Participants	13*	(a) Report numbers of individuals at each stage of study—eg numbers potentially eligible, examined for eligibility, confirmed eligible, included in the study, completing follow-up, and analysed<br>(b) Give reasons for non-participation at each stage<br>(c) Consider use of a flow diagram	5
Descriptive data	14*	(a) Give characteristics of study participants (eg demographic, clinical, social) and information on exposures and potential confounders<br>(b) Indicate number of participants with missing data for each variable of interest<br>(c) Summarise follow-up time (eg, average and total amount)	8-9
Outcome data	15*	Report numbers of outcome events or summary measures over time	8-13

For peer review only

For peer review only - http://bmjopen.bmj.com/site/about/guidelines.xhtml

Main results	16	(*a*) Give unadjusted estimates and, if applicable, confounder-adjusted estimates and their precision (eg, 95% confidence interval). Make clear which confounders were adjusted for and why they were included<br>(*b*) Report category boundaries when continuous variables were categorized<br>(*c*) If relevant, consider translating estimates of relative risk into absolute risk for a meaningful time period	NA
Other analyses	17	Report other analyses done—eg analyses of subgroups and interactions, and sensitivity analyses	NA
**Discussion**			
Key results	18	Summarise key results with reference to study objectives	14
Limitations	19	Discuss limitations of the study, taking into account sources of potential bias or imprecision. Discuss both direction and magnitude of any potential bias	17
Interpretation	20	Give a cautious overall interpretation of results considering objectives, limitations, multiplicity of analyses, results from similar studies, and other relevant evidence	14-17
Generalisability	21	Discuss the generalisability (external validity) of the study results	18
**Other information**			
Funding	22	Give the source of funding and the role of the funders for the present study and, if applicable, for the original study on which the present article is based	20

*Give information separately for exposed and unexposed groups.

Note: An Explanation and Elaboration article discusses each checklist item and gives methodological background and published examples of transparent reporting. The STROBE checklist is best used in conjunction with this article (freely available on the Web sites of PLoS Medicine at http://www.plosmedicine.org/, Annals of Internal Medicine at http://www.annals.org/, and Epidemiology at http://www.epidem.com/). Information on the STROBE Initiative is available at http://www.strobe-statement.org.

For peer review only

BMJ Open

Timing of adverse events in patients undergoing acute and elective hip arthroplasty surgery: a multi-centre cohort study

Journal:	*BMJ Open*
Manuscript ID	bmjopen-2022-064794.R1
Article Type:	Original research
Date Submitted by the Author:	08-Mar-2023
Complete List of Authors:	Magnéli, Martin; Karolinska Institute, Department of Clinical Sciences at Danderyd Hospital; Danderyd University Hospital, Department of Orthopaedics<br>Kelly-Pettersson, Paula ; Karolinska Institutet Department of Clinical Sciences Danderyd Hospital, Orthopaedics; Danderyd University Hospital, Department of orthopaedics<br>Rogmark, Cecilia; Lund University, Department of Clinical Sciences Malmö, Clinical and Molecular Osteoporosis Research Unit; Skåne University Hospital Malmö Orthopedics Clinic, Department of Orthopaedics<br>Gordon, Max; Karolinska Institute, Department of clinical sciences at Danderyd hospital; Danderyd University Hospital, Department of orthopaedics<br>Sköldenberg, Olof; Karolinska Institutet, Department of clinical sciences at Danderyd Hospital; Danderyd University Hospital, Department of orthopaedics<br>Unbeck, Maria; Hogskolan Dalarna; Karolinska Institutet, Department of clinical sciences at Danderyd Hospital
**Primary Subject Heading**:	Surgery
Secondary Subject Heading:	Epidemiology
Keywords:	Orthopaedic & trauma surgery < SURGERY, Adult orthopaedics < ORTHOPAEDIC & TRAUMA SURGERY, Hip < ORTHOPAEDIC & TRAUMA SURGERY

SCHOLARONE™
Manuscripts

I, the Submitting Author has the right to grant and does grant on behalf of all authors of the Work (as defined in the below author licence), an exclusive licence and/or a non-exclusive licence for contributions from authors who are: i) UK Crown employees; ii) where BMJ has agreed a CC-BY licence shall apply, and/or iii) in accordance with the terms applicable for US Federal Government officers or employees acting as part of their official duties; on a worldwide, perpetual, irrevocable, royalty-free basis to BMJ Publishing Group Ltd ("BMJ") its licensees and where the relevant Journal is co-owned by BMJ to the co-owners of the Journal, to publish the Work in this journal and any other BMJ products and to exploit all rights, as set out in our licence.

The Submitting Author accepts and understands that any supply made under these terms is made by BMJ to the Submitting Author unless you are acting as an employee on behalf of your employer or a postgraduate student of an affiliated institution which is paying any applicable article publishing charge ("APC") for Open Access articles. Where the Submitting Author wishes to make the Work available on an Open Access basis (and intends to pay the relevant APC), the terms of reuse of such Open Access shall be governed by a Creative Commons licence – details of these licences and which Creative Commons licence will apply to this Work are set out in our licence referred to above.

Other than as permitted in any relevant BMJ Author's Self Archiving Policies, I confirm this Work has not been accepted for publication elsewhere, is not being considered for publication elsewhere and does not duplicate material already published. I confirm all authors consent to publication of this Work and authorise the granting of this licence.

For peer review only

Timing of adverse events in patients undergoing acute and elective hip arthroplasty surgery: a multi-centre cohort study

Martin Magnéli[1,2*], Paula Kelly-Pettersson[1,2*], Cecilia Rogmark[3,4,5], Max Gordon[1,2], Olof Sköldenberg[1,2], Maria Unbeck[1,6]

[1] Karolinska Institutet, Department of Clinical Sciences, Danderyd Hospital, Unit of Orthopaedics, Stockholm, Sweden

[2] Danderyd University Hospital, Department of Orthopaedics, Stockholm, Sweden

[3] Skåne University Hospital, Department of Orthopaedics, Malmö, Sweden

[4] Lund University, Department of Clinical Sciences Malmö, Clinical and Molecular Osteoporosis Research Unit, Malmö, Sweden

[5] Swedish Arthroplasty Register, Centre of Registers Västra Götaland, Gothenburg, Sweden

[6] School of Health and Welfare, Dalarna University, Falun, Sweden

* Contributed equally

Corresponding author:
Martin Magnéli
Karolinska Institutet
Department of Clinical Sciences
Danderyd University Hospital
182 88 Stockholm
Sweden
martin.magneli@ki.se

Word count: 4235

For peer review only - http://bmjopen.bmj.com/site/about/guidelines.xhtml

ABSTRACT

Objective To explore timing in relation to all types of adverse events, severity and preventability for patients undergoing acute and elective hip arthroplasty.

Design A multi-centre cohort study using retrospective record review with Global Trigger Tool methodology in combination with data from several registers.

Setting 24 hospitals in 4 major regions of Sweden.

Participants Patients ≥18 years, undergoing acute or elective total or hemiarthroplasty of the hip were eligible for inclusion. Reviews of weighted samples of 1,998 randomly selected patient records were carried out using Global Trigger Tool methodology. The patients were followed for readmissions up to 90 days post-operatively throughout the whole country.

Results The cohort consisted of 667 acute and 1,331 elective patients. Most AEs occurred peri-and post-operatively (n=2093, 99.1%) and after discharge (n=1142, 54.1%). The median time from the day of surgery to the occurrence of AE was 8 days. The median days for different AE types ranged from 0 to 24.5 for acute and 0 to 71 for elective patients and peaked during different time periods. 40.2% of the AEs, both major and minor, occurred within post-operative day 0 to 5 and 86.9% of the AEs occurred within 30 days. Most of the AEs were deemed to be of major severity (n=1370, 65.5%) or preventable (n=1591, 76%).

Conclusions A wide variability was found regarding the timing of different adverse events with the majority occurring within 30 days. The timing and preventability varied regarding the severity. Most of the adverse events were deemed to be preventable and/or of major severity. To increase patient safety for patients undergoing hip arthroplasty surgery a better understanding of the multi-facetted nature of the timing of AEs in relation to the occurrence of differing AEs is needed.

For peer review only - http://bmjopen.bmj.com/site/about/guidelines.xhtml

Strengths and limitations of this study

- This is a registry-based national multicentre study including both acute and elective hip arthroplasty patients.
- Global Trigger Tool methodology for detecting adverse events was used for record review.
- All hospital admissions and unplanned outpatient visits occurring up to 90 days after surgery were reviewed.
- A weighted study sample optimised to select patients with adverse events was used to collect more data on adverse events compared with a random sample.

For peer review only

For peer review only - http://bmjopen.bmj.com/site/about/guidelines.xhtml

INTRODUCTION

Hip arthroplasty – "the operation of the century"[1] is an effective surgical treatment for both degenerative hip joint disease and femoral neck fractures. Still, adverse events (AEs) constituting a wide range of conditions can occur during the primary, index hospitalization and even years later. Both younger and older patients undergo hip arthroplasty nowadays, but the mean age of around 70 years indicates that a large group of elderly, and potentially frail patients undergo this surgery. This is particularly true for the group treated for femoral neck fractures.[2] AEs often entail suffering for the affected individual and the occurrence of preventable AEs reflects the gap between the actual care given and the expected standard of adequate safe care.[3 4]

Timing is an important factor in understanding the occurrence and prevention of AEs. It has been examined in some studies on arthroplasty surgery, but many of these included patients undergoing total primary hip or knee arthroplasty, and elective surgery was most common in these cohorts.[5-13] In contrast Bohl et al.[10] and Malik et al.[14], focused on geriatric patients undergoing hip arthroplasty after acute femoral neck fractures. Many of the timing studies[9-17] have used data from the American College of Surgeons National Surgical Quality Improvement Program with a follow-up period of 30 days post-operatively. This register does not include orthopaedic specific AEs such as dislocations.[9 12] Furthermore, many of these studies have had a rather narrow focus, for example, examining only peri-prosthetic joint infections[7], pulmonary embolism[8 18], venous thromboembolism[19], peri-prosthetic femur fractures[20], stroke[17 21], clostridium difficilie colitis[16], acute myocardial infarction[22 23] or a selection of pre-defined AEs.[5 9 10 12 14] In contrast, Parvizi et al.[6] and Yao et al.[11] have examined the occurrence of a broader range of AEs, but have not reported the timing for all the AEs that were identified.

There is a lack of knowledge regarding the timing of all types of AEs, both surgical and non-surgical, irrespective of whether they occur pre-, peri- or post-operatively in acute or elective procedures. . Furthermore, there is a need to examine timing in relation to the severity and preventability of AEs. Therefore, this study aimed to explore timing in relation to all types of adverse events, as well as severity and preventability in patients undergoing acute and elective hip arthroplasty.

For peer review only - http://bmjopen.bmj.com/site/about/guidelines.xhtml

METHODS

Study design

This sub-study is part of a retrospective multi-centre cohort study.[24] The aim of the main study was to validate the ability of a set of pre-defined ICD-10 codes used on a national level to compare hospitals following primary hip arthroplasties. The method and variables are the same for both the main study and the present study and are briefly described below. A more detailed description has been published previously.[24 25]

Participants and setting

The study population consisted of all patients aged ≥18 years undergoing either primary total hip arthroplasty or hemiarthroplasty, whether performed electively due to degenerative joint disease or acutely for a femoral neck fracture, that were entered into the SHAR during a three-year period (N=21,774). To increase the probability of selecting primary surgery admissions with the occurrence of at least one AE and to avoid excessive record review on admissions without AEs, we used a weighted sample. The study cohort was created by combining registry data from the Swedish Hip Arthroplasty Register (SHAR) and the National Patient Register. In total, 20 different selection groups for acute and elective arthroplasties were created.[24] The selection groups were based on the primary length of stay and readmission which were combined with patients that had pre-defined ICD-10 codes indicating AEs. Larger samples were drawn from the groups that had a high risk of AE (patients with extended length of stay, readmissions and AE ICD-10 codes).

The study cohort consisted of 2000 patients who had undergone either acute or elective primary surgery. The patients had their index surgery performed in hospitals located in one of four major regions in Sweden. Two patients were excluded leaving a final study cohort of 1998 patients. The patients were followed for a maximum of 90 days post-operatively for all in-patient and acute out-patient hospital care throughout the whole country, regardless of the location of the hospital where the index surgery was performed. Therefore, the study cohort had a total of 5423 admissions in 16 of the 21 regions in Sweden, with 69 hospitals involved, as individual patients may have had multiple admissions.

For peer review only - http://bmjopen.bmj.com/site/about/guidelines.xhtml

Data sources

This study used data from both medical records and three national registers, the National Patient Register, the Swedish Cause of Death Register, and the SHAR. In 2019, the SHAR had a completeness of 98% and 97% respectively for total hip replacements and hemiarthroplasties.[2] Data on primary surgery, such as date and type of surgery was obtained from the SHAR. This data was then cross-referenced with data from the National Patient Register using the Swedish personal identity number as a unique identifier. With this data, a timeline was created for each patient undergoing primary surgery and was used as a template to identify which admissions were to be reviewed.

We performed a manual retrospective record review using the Swedish version[26] of the Global Trigger Tool, a structured record review methodology.[27] This method involves a 2-stage review process.

Definitions

An AE was defined as suffering, physical harm or disease, as well as death related to the index admission and was not an inevitable consequence of the patient´s underlying disease or treatment.

A preventable adverse event was defined as an event that could have been prevented if adequate actions had been taken during the patient's contact with healthcare or social care.[28] A preventable event can be related to acts of commission or acts of omission.

The index admission was defined as the orthopaedic admission during which the patient had their primary hip arthroplasty surgery.

The occurrence date of the AE was defined as either the specific date when the sign or symptom first appeared or if this information was not available and the AE occurred after discharge the date when the patient contacted the care giver was then used. The occurrence date could also be the same date as the date of diagnosis.

Retrospective record review process

The record review process is described in detail elsewhere.[25] The recruitment of the ten reviewers (registered nurses, medical students, and physicians) was based on previous experience of record

For peer review only - http://bmjopen.bmj.com/site/about/guidelines.xhtml

review and/or convenience but all reviewers had previous experience of orthopaedic care.

A study manual, used as a complement to the Swedish Global Trigger Tool manual[26] with trigger definitions and descriptions, was created to clarify the study-specific interpretations and applications of triggers, definitions, and AE assessments. Triggers are clues or red flags in the medical record that indicate that a potential AE has occurred, for example, readmission within 30 days, re-operation.

The record review and time frame for the inclusion of an AE covered the period from the start of the admission to a maximum of 90 days post-operatively. During the follow-up period, it was possible to identify AEs in the records of patients receiving in-patient and/or out-patient care, irrespective of the speciality, at any hospital in Sweden.

The reviews were carried out in a 2-stage process using standardized data collection forms, one form for each review stage. In most cases, the same person carried out both stages of the review and the data was entered into a study-specific database.

In the first review stage, all record entries from all healthcare professionals were reviewed. The reviewers screened for the presence of one or more of the 38 pre-defined triggers in five modules of the Swedish version of the Global Trigger Tool.[26] Only records with triggers indicating at least one potential AE went forward to review stage 2.

In review stage 2, the reviewers sorted the different triggers into potential AEs, as more than one of the triggers can be involved in a single AE. Every potential AE was then reviewed separately. To qualify as an AE, a score of three or higher on a 4-point Likert scale was required (1=the AE was not related to index admission, 2=the AE was probably not related to index admission, 3=the AE was probably related to index admission, and 4=the AE was related to index admission). The preventability was also assessed using a similar scale with the same cut-off limit for inclusion regarding preventability.

Several other variables were collected, for example, the timing, type, and severity of the AEs. Severity was determined using a slightly modified version of the National Coordinating Council for Medication Error Reporting and Prevention (NCC MERP) Index.[29] NCC MERP Index categories E–I were included as they are related to harm i.e., AE. Events determined to be risks and no-harm incidents, i.e., NCC MERP categories A-D, were excluded.

For peer review only - http://bmjopen.bmj.com/site/about/guidelines.xhtml

Patient and public involvement

This study did not involve any patient or public representatives.

Statistical methods

Time from surgery to AE occurrence is presented in median days and interquartile range. The statistical analysis was performed using R 4.1.0 (R Project for Statistical Computing, Vienna, Austria), using tidyverse (v. 1.3.1) for dataset manipulation, ggplot2 (v. 3.3.5) for plots and htmlTable (v. 2.2.1) for creating tables.

RESULTS

Demographics

One third of the cohort were patients admitted acutely with a femoral neck fracture and two thirds underwent surgery on an elective basis. Patients with acute admissions were older, received hemiarthroplasties more often and were more often treated at university hospitals (table 1). Elective hemiarthroplasty represent a subgroup of patients, who all had the indication "Status post femoral neck fracture (FNF)" or avascular necrosis.

Table 1 Demographics

	**All patients** n= 1998	**Acute admitted** n= 667	**Elective admitted** n= 1331
Sex			
Female, n (%)	1250 (62.6)	444 (66.6)	806 (60.6)
Male, n (%)	748 (37.4)	223 (33.4)	525 (39.4)
Age, median years (min–max, IQR)	77 (18–100, 16)	84 (34–100, 10)	73 (18–99, 16)
LOS, median days (min–max, IQR)	6 (1–56, 5)	7 (1–56, 8)	5 (1–52, 3)
Type of surgery			
Total arthroplasty	1435 (71.8)	143 (21.4)	1292 (97.1)
Hemiarthroplasty	563 (28.2)	524 (78.6)	39 (2.9)
Type of hospital, n (%)			
University	630 (31.5)	295 (44.2)	335 (25.2)
Central County Council	556 (27.8)	180 (27.0)	376 (28.2)
County Council	531 (26.6)	109 (16.3)	422 (31.7)
Private*	281 (14.1)	83 (12.4)	198 (14.9)

LOS, length of stay, IQR, interquartile range.
* Most of the surgeries are publicly financed through agreements with the regional authorities.
Note: Weighted samples, the values are not representative for average Swedish orthopaedic care concerning hip arthroplasty.

For peer review only - http://bmjopen.bmj.com/site/about/guidelines.xhtml

Adverse event outcomes

In total, 2116 AEs of varying severity were identified in 1171 (58.6%) patients in our weighted sample of which 527 (45.0%) of these patients were affected by more than one AE. The patients admitted acutely sustained 981 (46.4%; min to max, 1–10) AEs and the elective patients 1135 (53.6%; min to max 1–7). Acute patients were affected by an AE to a higher extent compared to elective patients, 71.4% (476/667) versus 52.3% (696/1331). The patients in the cohort had in total 3425 all-cause re-admissions during the follow-up period. However, some of the re-admissions were not related to the index admission and were therefore not considered to be related to an AE.

Timing overall and in connection to adverse event types

Four AEs did not have a correct date registered and were excluded, leaving 2112 AEs remaining for the timing analysis, 980 AEs in acute patients and 1132 AEs in elective patients. Nineteen (0.9%) AEs occurred from admission to the day before surgery and 2093 (99.1%) AEs occurred from the day of surgery, including peri-operative AEs, up to 90 days post-operatively.

Of the 2112 AEs, 970 (45.9%) occurred during the index orthopaedic admission, the remaining 1142 (54.1%) AEs occurred after discharge. The AEs occurring after discharged were distributed as follow: 866 (41.0%) within 30 days and 276 (13.1%) between 31 and 90 days after surgery. Of the patients undergoing acute surgery 486 (49.6%) of the 980 AEs occurred during the index admission, compared to elective patients where 484 (42.8%) of 1132 AEs occurred during the index admission.

The median time from day of surgery to an AE was 8 days for the 2093 AEs occurring on the day of surgery and post-operatively. The corresponding median for both acute and elective patients was also 8 days (summary in table 2, full table in supplementary material).

For both acute and elective patients, pressure ulcers, skin, tissue and superficial vessel harm, peri- and post-operative bleeding/haematomas not requiring re-operation and pneumonia were common AEs and peaked during the index admission. Within 30 days after surgery, but after discharge, dislocation of the prothesis and infections such as deep peri-prosthetic infections, superficial wound infections and urinary tract infections were commonly occurring AEs and peaked in this period for both groups. No AE peaked after 30 days post-operatively for the acute patients, whereas for the elective patients, pulmonary embolisms, deep venous thromboses, mechanical

For peer review only - http://bmjopen.bmj.com/site/about/guidelines.xhtml

complications and surgical harm – other all peaked after 30 days. Some types of AEs continued to occur at a high rate such as dislocations in both groups after 30 days (table 2).

For peer review only

For peer review only - http://bmjopen.bmj.com/site/about/guidelines.xhtml

Table 2 Summary of identified adverse events for acute respective elective patients, sorted by descending order of types of AEs for all patients. Full table available in supplementary material.

	**All**		**Acute**		**Elective**	
**Type of AEs**	**N (%)**	**Median time from surgery to AE (days)**	**n (%)**	**Median time from surgery to AE (days)**	**n (%)**	**Median time from surgery to AE (days)**
Dislocation of the prothesis	274 (13.1)	23	101 (10.4)	23	173 (15.4)	23
Pressure ulcer	189 (9)	5	143 (14.8)	5	46 (4.1)	4.5
Urinary tract infection	163 (7.8)	8	93 (9.6)	9	70 (6.2)	8
Peri-prosthetic joint infection	149 (7.1)	20	36 (3.7)	19	113 (10)	20
Superficial wound infection	147 (7)	11	59 (6.1)	11	88 (7.8)	13
Skin. tissue and superficial vessel harm[1]	123 (5.9)	4	78 (8.1)	5	45 (4)	4
Peri-/post-operative bleeding/ haematoma - did not require re-operation	117 (5.6)	0	29 (3)	3	88 (7.8)	0
Pneumonia	117 (5.6)	4	77 (8)	3	40 (3.6)	5
Falls	87 (4.2)	15	38 (3.9)	19	49 (4.4)	10
Neurological[2]	87 (4.2)	2	46 (4.8)	2.5	41 (3.6)	2
Distended urinary bladder	81 (3.9)	3	52 (5.4)	3	29 (2.6)	3
Pulmonary embolism	64 (3.1)	27	15 (1.5)	12	49 (4.4)	32
Gastric ulcer	45 (2.2)	6	19 (2)	7	26 (2.3)	5.5
Cardiovascular[3]	40 (1.9)	3	14 (1.4)	13.5	26 (2.3)	3
Pain	36 (1.7)	4	7 (0.7)	3	29 (2.6)	9
Gastro-intestinal[4]	31 (1.5)	7	9 (0.9)	7	22 (2)	6.5
Peri-operative fracture	31 (1.5)	0	8 (0.8)	0	23 (2)	0
Renal failure	31 (1.5)	2	20 (2.1)	2	11 (1)	3
Deep vein thrombosis	29 (1.4)	42	10 (1)	24.5	19 (1.7)	46
Allergic reaction	25 (1.2)	5	8 (0.8)	5	17 (1.5)	4
Leg length difference	23 (1.1)	3	4 (0.4)	2.5	19 (1.7)	3
Gastrointestinal infection	22 (1.1)	9	17 (1.8)	11	5 (0.4)	4
Septicaemia	20 (1)	17.5	14 (1.4)	17.5	6 (0.5)	13.5
Mechanical complication[5]	20 (1)	25	7 (0.7)	24	13 (1.2)	29
Unclear infection	17 (0.8)	5	11 (1.1)	5	6 (0.5)	4
Respiratory	17 (0.8)	1	9 (0.9)	1	8 (0.7)	1
Myocardial infarction	15 (0.7)	7	6 (0.6)	3.5	9 (0.8)	8
Mouth and throat infection	12 (0.6)	9	8 (0.8)	9	4 (0.4)	10
Peripheral nerve injury[6]	12 (0.6)	2	3 (0.3)	8	9 (0.8)	2
Ileus	9 (0.4)	5	4 (0.4)	6	5 (0.4)	4
Stroke	9 (0.4)	4	4 (0.4)	5	5 (0.4)	4
Electrolyte imbalance	8 (0.4)	5.5	1 (0.1)	5	7 (0.6)	6
AE caused by anaesthesia[7]	7 (0.3)	1	3 (0.3)	0	4 (0.4)	1
Malnutrition	7 (0.3)	6	4 (0.4)	13	3 (0.3)	6
Bleeding - not related to surgery[8]	5 (0.2)	11	2 (0.2)	6.5	3 (0.3)	11
Infection other	5 (0.2)	11	4 (0.4)	11	1 (0.1)	6
Bleeding - that required re-operation	5 (0.2)	8	1 (0.1)	8	4 (0.4)	5

For peer review only - http://bmjopen.bmj.com/site/about/guidelines.xhtml

Surgical harm – other	3 (0.1)	71	0 (0)	NA	3 (0.3)	71
Multi-organ failure	1 (0)	3	1 (0.1)	3	0 (0)	NA
Other	10 (0.5)	7.5	3 (0.3)	19	7 (0.6)	7
Total	2093 (100)	8	968 (100)	8	1125 (100)	8

AE, adverse event.
Examples of adverse events in these types; [1] blister, extravasation, phlebitis; [2] acute confusion, hallucination, lethargy; [3] heart failure; [4] obstipation, vomiting, diarrhoea; [5] fracture without a fall, re-operation after several dislocations; [6] foot drop; [7] awareness, aspiration; [8] in connection to urinary catheter or warfarin treatment.

The 8 most common types of AEs and their median day of occurrence are shown in figure 1. Of these, peri-/post-operative bleeding/haematoma had the earliest day of occurrence at 3 (interquartile range, 12) days for acute patients and 0 (7) days for elective patients. The second earliest occurring AE was skin, tissue and superficial vessel harm with a median of 5 (9) for acute and 4 (4) days for the elective patients. Dislocations had the longest median time to occurrence with 23 (24 versus 29) days for both groups.

Add figure 1 here.

Timing outcomes within 5 days post-operatively

In total, 40.2% of both minor and major AEs (n=842/2093) occurred either on the day of surgery or during the following 5 post-operative days (table 3). Some types of AEs were only common early in the 5-day time frame, for example, peri-operative haemorrhage, peri-operative fractures, and dislocation on post-operative day 1 while some other AEs such as skin, tissue and superficial vessel harm and distended urinary bladder were frequent from the day of surgery and onwards. Later in the time frame, infections such as pneumonia, urinary tract infections and superficial wound infections began to occur more commonly.

Table 3 Peri-operative and early post-operative adverse events (AEs), the 5 most common AE types per day

Type of AE, n	POD 0	POD 1	POD 2	POD 3	POD 4	POD 5
Peri-operative haemorrhage	59					
Peri-operative fracture	25					
Skin, tissue and superficial vessel harm	11	14		17	10	11
Neurological	9	20	18			
Distended urinary bladder	8	9	16	17	8	
Dislocation		14				
Pressure ulcer		14	21	30	17	15
Pneumonia			27	18		7
Urinary tract infection			14	11	13	5
Superficial wound infection					12	5

For peer review only - http://bmjopen.bmj.com/site/about/guidelines.xhtml

Total for the 5 most common per day, (%) of total per day	112 (64.0)	71 (48.6)	96 (56.8)	93 (58.5)	60 (55.0)	43 (51.2)
Total for all AEs per day	175	146	169	159	109	84
POD, post-operative day.						

Timing and preventability in relation to severity

Preventability and timing varied within and between the acute and elective groups, as well as, for the different AE types regarding severity. Most of the AEs were deemed to be of major severity (n=1370, 65.5%, NCC MERP categories F-I) or preventable (n=1591, 76.0%). The proportion of major AEs was higher among the elective patients compared to the acute, 72.9% versus 56.8%. In contrast, the proportion of AEs that contributed to death was higher among the acute patients, 3.9% versus 0.5%. All deaths in the elective group were deemed preventable and the AEs contributing to death began to occur later (median days 20.5) in contrast to the 65.9% preventable AEs for the acute group with a median of 15 days (table 4). Median days to death was in total 29 days for 28 acute patients and 25 days for 4 elective patients. Examples of AEs resulting in some degree of permanent harm, classified according to the Swedish patient insurance assessment standards, were dislocations, deep peri-prosthetic infections, fractures, thromboses/embolisms, and leg length difference.

In total, a third of the AEs were classified as minor (NCC MERP category E) including, for example, pressure ulcers, urinary tract infections, skin harm, falls with minor injury, distended urinary bladders, and intravenous infiltrations. In total, minor AEs and AEs that required intervention necessary to sustain life within 60 minutes (NCC MERP category H) occurred earlier (median 4 respective 1 day) compared to the other NCC MERP categories. Among the acute patients, minor AEs occurred more often, although somewhat later and were assessed to be more preventable compared those in elective patients (table 4).

For peer review only

Table 4 Number, proportion, and timing of adverse events (AEs) and preventable AEs in relation to severity

	**Severity according to NCC MERP**					
Groups	E	F	G	H	I	Total
**Adverse events, all patients**						
N (%) of AEs	723 (34.5)	794 (37.9)	518 (24.7)	14 (0.7)	44 (2.1)	2093 (100)
Median (IQR) days	4 (7)	8 (15)	21 (27)	1 (3)	15.5 (18.75)	8 (16)
N (%) of preventable AEs	528 (73.0)	541 (68.1)	482 (93.0)	11 (78.6)	29 (65.9)	1,591 (76.0)
Median (IQR), days, preventable AEs	4 (7)	8 (14)	21 (27)	1 (2.5)	16 (18)	9 (17)
**Adverse events, acute patients**						
N (%) of AEs	418 (43.2)	335 (34.6)	169 (17.5)	8 (0.8)	38 (3.9)	968 (100)
Median (IQR) days	5 (8)	7 (13)	22 (23)	1 (1.75)	15 (20.25)	8 (14)
N (%) of preventable AEs	321 (76.8)	243 (72.5)	155 (91.7)	8 (100)	23 (60.5)	750 (77.5)
Median (IQR), days, preventable AEs	5 (7)	7 (12.5)	22 (20.5)	1 (1.75)	15 (19.5)	8 (14.75)
**Adverse events, elective patients**						
N (%) of AEs	305 (27.1)	459 (40.8)	349 (31.0)	6 (0.5)	6 (0.5)	1125 (100)

Median (IQR) days	3 (6)	8 (16)	21 (31)	1.5 (6.25)	20.5 (13)	8 (18)
N (%) of preventable AEs	207 (67.9)	298 (64.9)	327 (93.7)	3 (50.0)	6 (100)	841 (74.8)
Median (IQR), days, preventable AEs	3 (5)	8.5 (15.75)	21 (29)	1 (14.5)	20.5 (13)	10 (19)

NCC MERP, National Coordinating Council for Medication Error Reporting and Prevention Index.
E, Contributed to or resulted in temporary harm; F, Contributed to or resulted in temporary harm that prolonged hospitalization, required out-patient care or readmission; G, Contributed to or resulted in permanent patient harm; H, Required intervention necessary to sustain life within 60 minutes; I, Contributed or resulted in the patient's death
IQR, interquartile range.

For peer review only

For peer review only - http://bmjopen.bmj.com/site/about/guidelines.xhtml

DISCUSSION

To the best of our knowledge, this is the first study to explore all types of AEs with associated timing data, both in total and separately for acute and elective patients. Most of the AEs occurred after the index admission in both groups. As much as 87% of the AEs occurred within 30 days post-operatively. The overall median time of 8 days from the day of surgery to an AE was the same for both acute and elective patients. Pressure ulcers and skin, tissue and superficial vessel harm were common and peaked for both group during the index admission. Dislocations and deep peri-prosthetic joint infections were common reasons for readmissions within 30 days after surgery and peaked during this time frame. Dislocations continued to occur to a high extent after 30 days. Forty percent of all AEs, both major and minor, occurred from the day of surgery and up to and including the fifth post-operative day. The timing and preventability varied regarding the severity. Most of the AEs were deemed to be preventable and/or of major severity.

Previous timing studies examining patients undergoing arthroplasty have used varying data collection methods, included varying types of arthroplasty surgery, often with a focus on total hip replacements in elective patients, and the criteria for AEs, timing and follow-up periods differ. Furthermore, some studies do not report data for hip arthroplasty separately. This makes comparison of the outcomes somewhat difficult.

Type of adverse events and timing in general

A wide variability was found regarding the timing of AEs. Peri-/post-operative bleeding/ haematoma, peri-operative fracture, AEs caused by anaesthesia, respiratory AEs, renal failure, peripheral nerve injuries and neurological AEs occurred early, within the first 2 days post-operatively. The latest occurring AEs included surgical harms of other types, deep vein thromboses, mechanical complications, dislocations and peri-prosthetic infections which ranged from median 20 to 71 days. Some AEs continued to occur up until the end of the follow-up period.

Dislocation was the most common AE in elective patients and the second most common in acute patients. It occurred at a median of 3 weeks and in 9 of 10 cases after discharge. This AE is seldom reported in timing studies, for example, the commonly used National Surgical Quality Improvement Program does not include dislocations and other orthopaedic-specific AEs.[9 12] However, Ali et al.[30] also found that dislocation was one of the most common AEs which clustered early after discharge. In their study, after 30 days post-operatively 74% of the

For peer review only - http://bmjopen.bmj.com/site/about/guidelines.xhtml

dislocations requiring readmission had occurred and this was higher than the 51% in the present study. Even after 30 days, dislocations continued to occur at a high rate.

Other common AEs were infections such as peri-prosthetic, urinary tract and superficial wound infections, the majority of which occurred after discharge. The median time to superficial wound infections and peri-prosthetic infections was 11 versus 20 days and the corresponding for Malik et al.[14] was 16 versus 23 days. Bohl et al.[9 10] reported in two studies surgical site infections with median days of 17 and 16, respectively. Our median time to urinary tract infections is in line with results from other studies with a range of 7 to 8 days.[9 10 14]

In contrast to other AEs, pulmonary embolism and deep vein thrombosis occurred relatively late at 4 and 6 weeks respectively. The longer time to the occurrence of deep vein thrombosis compared to pulmonary embolism can also be seen in other studies.[5 9 10 14] In contrast, these two AE types occurred later in our cohort than in other studies where median days to pulmonary embolism and deep vein thrombosis ranged from 3-5 days and 6-9 days, respectively.[5 9 10 14] A partial explanation can be that we had a longer follow-up period which affects the median day outcomes.

Several of our most common AE types, such as, pressure ulcers, skin, tissue and superficial vessel harm, falls, neurological harm, and distended urinary bladder are not covered by similar timing studies examining AEs in hip arthroplasty patients. These AEs, which mostly peaked during the index admission, were often classified as minor and preventable but not directly related to the surgical intervention. Nevertheless, minor AEs may also lead to suffering for the patient. To reduce the number of AEs occurring in orthopaedic care, it is important to analyse AEs also from a timing perspective and work proactively pre-, peri- and post-operatively in interprofessional teams both inside and outside the operating room.[31]

Two timing studies focused on geriatric patients with hip fractures in general[14] and those treated with arthroplasty.[10] Although, timing of specific AEs was quite similar, pulmonary embolism, deep vein thrombosis and sepsis occurred somewhat later and superficial wound infections occurred somewhat earlier in our acute cohort. The patterns of AE types in relation to early or late onset were similar for the most part.

Timing during and after index admission, early and late onset

We found that less than half of all AEs occurred during the index admission. The median length of index stay is longer in our study compared to several other studies. This can be explained by

For peer review only - http://bmjopen.bmj.com/site/about/guidelines.xhtml

the fact that we included acute patients and admissions with longer length of stay were over-represented due to the sampling technique used in the main study. Nevertheless, other studies with shorter length of stay found that most of the AEs occurred during the index admission.[9 10 12] In contrast, Yao et al.[11] had a higher AE occurrence after discharge when studying a cohort of home-admitted patients. As we are moving toward shorter hospital stays and an increased amount of day-surgery, the risk of major AEs occurring outside the hospitals increases. Therefore, a careful follow-up plan after discharge can be of value. Co-production of healthcare outcomes by the healthcare professionals and the patient as an important participating partner may be a significant intervention in the preventive work to reduce the occurrence of AEs and increase patient safety.[32] The patients, and in some cases their significant others and the staff at nursing homes, need to be well informed regarding which signs and symptoms of AEs to be aware of and how to manage self-care. These are central aspects during the rehabilitation period.

Most of the AEs in the present study occurred within 30 days post-operatively. Many other studies had a follow-up period of only 30 days and most of the AEs would probably be detected during this period. However, this time frame may lead to a risk of underestimation of the incidence of AEs and a bias towards the earlier-diagnosed AEs as some AE types, for example, surgical site infections did not plateau within this specific follow-up period.[9 10 14]

Nearly half of all AEs occurred within the first 5 days post-operatively. Still, none of the 4 “catastrophic” AEs, pre-defined by Johnson et al.[12] namely pulmonary embolism, myocardial infarction or cardiac arrest, cerebrovascular accident, or death were among our 5 most common AEs during this time period. The corresponding results for Parvizi et al.[6] was 93% for life-threating medical AEs. Belmont et al.´s[13] findings showed that 77% of total hip arthroplasty patients who had an adverse cardiac event in the form of myocardial infarction or cardiac arrest experienced it within 3 days after surgery.

Severity and preventability

The timing and preventability varied regarding the severity for the acute and elective patients. We assessed severity with a widely used scale in AE studies using the Global Trigger Tool record review methodology.[27] The severity of each AE was assessed independently, and a specific type of AE, for example pressure ulcers, falls and urinary tract infections could be assessed as having varying degrees of severity depending on the harm caused. Parvizi et al.[6] and Yao et al.[11] classified all, for example, pneumonia as minor AEs while we classified them

For peer review only - http://bmjopen.bmj.com/site/about/guidelines.xhtml

as major. On the other hand, Parvizi et al.[6] defined AEs that resulted in, for example, prolonged hospital stays as minor while we classified these AEs as major (NCC MERP F).

In contrast to other studies[5 6 11-14], we did not include death as a separate AE type as we considered death to be the consequence of an AE and belongs to the severity classification. The median time to death was around 4 weeks for both groups. For some patients more than one AE may have contributed to their death (NCC MERP category I).

Further research is needed to explore the multi-facetted nature of the timing in relation to the occurrence of minor and major AEs, both surgical and non-surgical, during the pre-, peri- or post-operative periods of patient care. A multi-pronged approach is required in the prevention of AEs[14], as most AEs were deemed to be preventable, and patients were commonly affected by more than one AE. To increase patient safety for this patient group, additional research is needed regarding the implementation and evaluation of timely targeted interventions during the varying phases of the patient-care process, focusing on preventable AEs.

Limitations and strengths

The study has several limitations. We have not included AEs occurring after 90 days post-operatively. Using record review as the data collection method may have led to an underestimation of AEs due to underreporting. Furthermore, AEs detected and treated outside the hospital setting may have been missed. These are probably minor AEs as severe AEs would be more likely to be treated in a hospital setting. This has led to severe outcome, for example, peri-prosthetic joint infections being more common compared to superficial wound infections. Even though we reported the time to occurrence in most cases, some AEs occurring after discharge may have had a delayed timing date if the patient was not specific enough about when the first symptom of the AE occurred. In these cases, the date when the patient first contacted the care giver was used. The weighted study sample used in this study was useful for maximising the inclusion of patients with an AE in relation to the main study´s aim and this is the explanation for the high AE rates. This is not the incidence of AEs in the source population. The estimated incidence for different AEs has been calculated by adjusting for the sampling weights in previous publications from this study.[24]

The multi-centre design with a wide range of patients of all ages and types of hospitals can be considered as a strength. The 90-day national follow-up, regardless of index hospital should be sufficient to detect most acute, sub-acute and rare AEs. To ensure the reliability and validity of

For peer review only - http://bmjopen.bmj.com/site/about/guidelines.xhtml

our data, the study has been closely monitored to check the correctness and completeness of the data, enabling for a good control of the review process. We used a stringent definition of what constitutes an AE and attained very good kappa values in the review process[24]. We only included AEs related to the care given and excluded conditions judged to be related to an underlying disease or condition, unless the disease or condition deteriorated due to healthcare. We believe our results to be valid and generalizable at least to Western world patients and/or publicly financed healthcare.

For peer review only

For peer review only - http://bmjopen.bmj.com/site/about/guidelines.xhtml

Acknowledgements

The authors wish to thank Marie Ax, Susanne Hansson, Zara Hedlund, Mirta Stupin, Tim Hansson, Lovisa Hult-Ericson, and Christina Jansson for their help and support with the review records. We would also like to thank all departmental managers for providing access to the patient records and Per Nydert for his help with the study database.

Contributors

MM collected data, analysed the data, and critically revised the manuscript.
PKP interpreted the data and contributed to the drafting of the work.
CR contributed to the design of the study and contributed to the drafting of the work.
MG contributed to the design of the study, collected data, and critically revised the manuscript.
OS contributed to the design of the study, collected data, and critically revised the manuscript.
MU contributed to the design of the study, monitored the reviews, analysed and interpreted the data, and was mainly responsible for the drafting of the work.
All authors have approved the final version of the manuscript and agree to be accountable for all aspects of the work.

Competing interests

None to declare.

Data sharing statement

No additional data is available.

Ethical approval

Ethical approval was provided by the Regional Ethics Committee of Gothenburg (516-13 and T732-13). Permission for data access for the reviewers was granted by the head of each respective unit. The patients did not provide an informed consent to the record review.

For peer review only

Funding

This work was supported by the Swedish Patient Insurance. The funder has not been involved in any part of the study, in either writing the manuscript or the decision to submit the manuscript for publication.

Patient and Public involvement

Patients and/or the public were not involved in the design, conduct, reporting or dissemination plans of our research.

Patient consent for publication

Not applicable.

ORCHID IDs

Martin Magnéli https://orchid.org/ 0000-0003-0341-0227

Paula Kelly-Pettersson https://orchid.org/0000-0003-3685-2953

Cecilia Rogmark https://orchid.org/0000-0002-6556-4320

Max Gordon https://orchid.org/ 0000-0002-8080-5815

Olof Sköldenberg https://orchid.org/ 0000-0003-4155-8425

Maria Unbeck https://orchid.org/ 0000-0002-5090-0352

For peer review only

REFERENCES

1 Learmonth ID, Young C, Rorabeck C. The operation of the century: total hip replacement. *Lancet* 2007;370:1508-19.

2 W-Dahl A, Kärrholm J, Nauclér E, *et al*. Annual report 2021. The Swedish Arthroplasty Register. Available: https://registercentrum.blob.core.windows.net/slr/r/SAR-Annual-Report-2021-SJlAFmlRl5.pdf [Accessed 10 May 2022].

3 Duarte Sda C, Stipp MA, da Silva MM, *et al*. Adverse events and safety in nursing care. *Rev Bras Enferm* 2015;68:136-46, 144-54.

4 Pettersson PK, Skoldenberg O, Samuelsson B, *et al*. The identification of adverse events in hip fracture patients using the Global Trigger Tool: A prospective observational cohort study. *Int J Orthop Trauma Nurs* 2020;38:100779.

5 Mantilla CB, Horlocker TT, Schroeder DR, *et al*. Frequency of myocardial infarction, pulmonary embolism, deep venous thrombosis, and death following primary hip or knee arthroplasty. *Anesthesiology* 2002;96:1140-6.

6 Parvizi J, Mui A, Purtill JJ, *et al*. Total joint arthroplasty: When do fatal or near-fatal complications occur? *J Bone Joint Surg Am* 2007;89a:27-32.

7 Pulido L, Ghanem E, Joshi A, *et al*. Periprosthetic joint infection: The incidence, timing, and predisposing factors. *Clin Ortho Relat Res* 2008;466:1710-15.

8 Parvizi J, Huang R, Raphael IJ, et al. Timing of symptomatic pulmonary embolism with warfarin following arthroplasty. *J Arthroplasty* 2015;30:1050-3.

9 Bohl DD, Ondeck NT, Basques BA, *et al*. What is the timing of general health adverse events that occur after total joint arthroplasty? *Clin Orthop Relat Res* 2017;475:2952-59.

10 Bohl DD, Samuel AM, Webb ML, *et al*. Timing of adverse events following geriatric hip fracture surgery: A Study of 19,873 patients in the American college of surgeons national surgical quality improvement program. *Am J Orthop (Belle Mead NJ)* 2018;47.

11 Yao DH, Keswani A, Shah CK, *et al*. Home discharge after primary elective total joint arthroplasty: postdischarge complication timing and risk factor analysis. *J Arthroplasty* 2017;32:375-80.

12 Johnson DJ, Hartwell MJ, Weiner JA, *et al*. Which postoperative day after total joint arthroplasty are catastrophic events most likely to occur? *J Arthroplasty* 2019;34:2466-72.

13 Belmont PJ, Goodman GP, Kusnezov NA, *et al*. Postoperative myocardial infarction and cardiac arrest following primary total knee and hip arthroplasty: rates, risk factors, and time of occurrence. *J Bone Joint Surg Am* 2014;96a:2025-31.

For peer review only - http://bmjopen.bmj.com/site/about/guidelines.xhtml

14 Malik AT, Quatman CE, Phieffer LS, *et al*. Timing of complications following surgery for geriatric hip fractures. *J Clin Orthop Trauma* 2019;10:904-11.

15 Minhas SV, Kester BS, Lovecchio FC, et al. Nationwide 30-day readmissions after elective orthopedic surgery: reasons and implications. *J Healthc Qual* 2017;39:34-42.

16 Bovonratwet P, Bohl DD, Malpani R, *et al*. Incidence, risk factors, and impact of clostridium difficile colitis following primary total hip and knee arthroplasty. *J Arthroplasty* 2018;33:205-10.e1.

17 Haynes MS, Alder KD, Bellamkonda K, *et al*. Incidence, predictors, and timing of post-operative stroke following elective total hip arthroplasty and total knee arthroplasty. *PLoS One* 2020;15:e0239239.

18 Hu C, Liu C, Wang Y, *et al*. The timing of symptomatic pulmonary embolism in patients with nonwarfarin anticoagulants following elective primary total joint arthroplasty. *J Arthroplasty* 2020;35:1703-07.

19 Shohat N, Goel R, Ludwick L, *et al*. Time to venous thromboembolism events following total hip arthroplasty: A comparison between aspirin and warfarin. *J Arthroplasty* 2022. [Published Online First: 2022/02/13]

20 Lygrisse KA, Gaukhman GD, Teo G, et al. Is surgical approach for primary total hip arthroplasty associated with timing, incidence, and characteristics of periprosthetic femur fractures? *J Arthroplasty* 2021;36:3305-11.

21 Lalmohamed A, Vestergaard P, Cooper C, *et al*. Timing of stroke in patients undergoing total hip replacement and matched controls: a nationwide cohort study. *Stroke* 2012;43:3225-9.

22 Lalmohamed A, Vestergaard P, Klop C, *et al*. Timing of acute myocardial infarction in patients undergoing total hip or knee replacement: a nationwide cohort study. *Arch Intern Med* 2012;172:1229-35.

23 Gandhi R, Petruccelli D, Devereaux PJ, et al. Incidence and timing of myocardial infarction after total joint arthroplasty. *J Arthroplasty* 2006;21:874-7.

24 Magnéli M, Unbeck M, Rogmark C, et al. Validation of adverse events after hip arthroplasty: a Swedish multi-centre cohort study. *BMJ Open* 2019;9:e023773.

25 Hommel A, Magneli M, Samuelsson B, et al. Exploring the incidence and nature of nursing-sensitive orthopaedic adverse events: A multicenter cohort study using Global Trigger Tool. *Int J Nurs Stud* 2020;102:103473.

For peer review only - http://bmjopen.bmj.com/site/about/guidelines.xhtml

26 SALAR. *Marker-based record review: to identify and measure harm in healthcare* [in Swedish: Markörbaserad journalgranskning: för att identifiera och mäta skador i vården]. Stockholm: Swedish Association of Local Authorities and Regions (SALAR), 2012.

27 Griffin FA, Resar, R.K. *IHI Global Trigger Tool for measuring adverse events* (second edition). IHI Innovation Series white paper. Cambridge, MA: Institute for Healthcare Improvement, 2009.

28 Swedish Codes of Statutes. Patient Safety Act (2010:659) [In Swedish: Patientsäkerhetslag 2010:659]. Available: https://www.riksdagen.se/sv/dokument-lagar/dokument/svensk-forfattningssamling/patientsakerhetslag-2010659_sfs-2010-659 [Accessed 10 May 2022].

29 National Coordinating Council for Medication Error Reporting and Prevention. Available: https://www.nccmerp.org/types-medication-errors [Accessed 10 May 2022].

30 Ali AM, Loeffler MD, Aylin P, *et al*. Timing of readmissions after elective total hip and knee arthroplasty: Does a 30-day all-cause rate capture surgically relevant readmissions? *J Arthroplasty* 2021;36:728-33.

31 Unbeck M, Dalen N, Muren O, *et al*. Healthcare processes must be improved to reduce the occurrence of orthopaedic adverse events. *Scand J Caring Sci* 2010;24:671-7.

32 World Health Organization. Exploring patient participartion in reducing health-care-related safety risks. 2013. Available: https://www.euro.who.int/__data/assets/pdf_file/0010/185779/e96814.pdf [Accessed 29 April 2022].

For peer review only - http://bmjopen.bmj.com/site/about/guidelines.xhtml

Legends

Figure 1. Median days from the day of surgery for the 8 most common types of adverse events up to 90 days post-operatively.

For peer review only

For peer review only - http://bmjopen.bmj.com/site/about/guidelines.xhtml

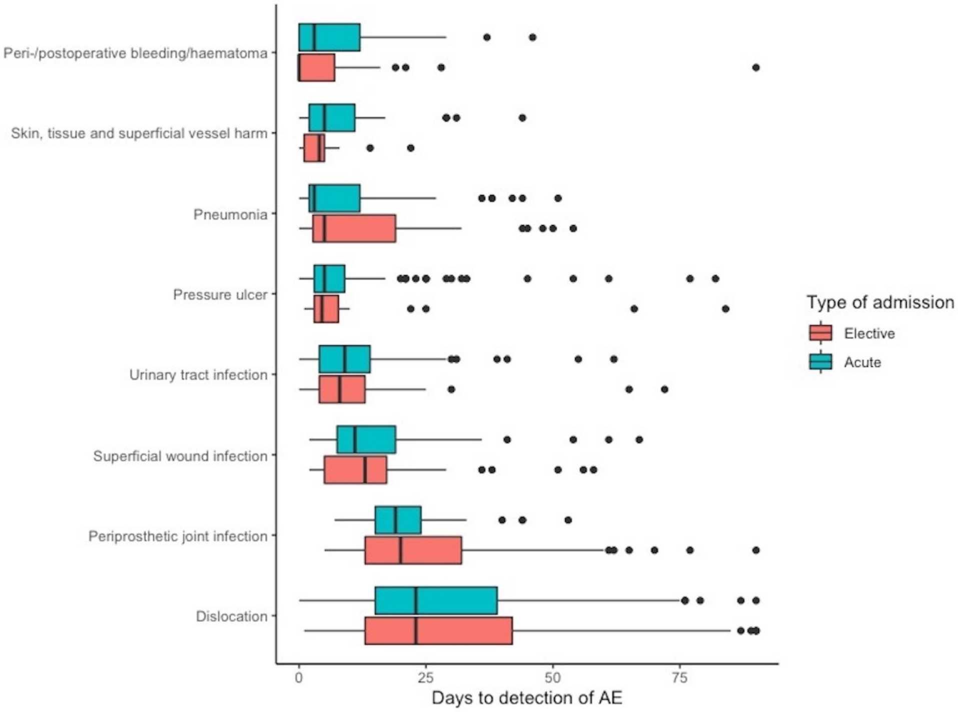

Figure 1 Median days from the day of surgery for the 8 most common types of adverse events up to 90 days post-operatively. The median (interquartile) day of occurrence for acute respective elective patients was for peri- or post-operative bleeding/haematoma 3 (7), 0 (12), skin, tissue and superficial vessel harm 5 (9), 4 (4), pneumonia 3 (10), 5 (16.25), pressure ulcer 5 (6), 4.5 (4.75), urinary tract infection 9 (10), 8 (9), superficial wound infection 11 (11.5), 12 (11.75), peri-prosthetic joint infection 19 (9), 20 (19), and dislocation 23 (24), 23 (29).

76x57mm (400 x 400 DPI)

Table 2 Overview of identified adverse events for acute respective elective patients during different time frames and time to occurrence, sorted by descending order of types of AEs for all patients

	All		Acute					Elective				
**Type of AEs**	**N (%)**	**Median time from surgery to AE (days)**	**n (%)**	**Median time from surgery to AE (days)**	**Occurred during index admission n (%)**	**Occurred within 30 days but after index n (%)**	**Occurred within 31–90 days n (%)**	**n (%)**	**Median time from surgery to AE (days)**	**Occurred during index admission n (%)**	**Occurred within 30 days but after index n (%)**	**Occurred within 31–90 days n (%)**
Dislocation of the prothesis	274 (13.1)	23	101 (10.4)	23	11 (2.3)	56 (14)	34 (36.6)	173 (15.4)	23	23 (4.8)	83 (17.8)	67 (36.6)
Pressure ulcer	189 (9)	5	143 (14.8)	5	91 (19.1)	46 (11.5)	6 (6.5)	46 (4.1)	4.5	33 (6.9)	11 (2.4)	2 (1.1)
Urinary tract infection	163 (7.8)	8	93 (9.6)	9	39 (8.2)	49 (12.3)	5 (5.4)	70 (6.2)	8	32 (6.7)	36 (7.7)	2 (1.1)
Peri-prosthetic joint infection	149 (7.1)	20	36 (3.7)	19	4 (0.8)	27 (6.8)	5 (5.4)	113 (10)	20	4 (0.8)	80 (17.2)	29 (15.8)
Superficial wound infection	147 (7)	11	59 (6.1)	11	22 (4.6)	32 (8)	5 (5.4)	88 (7.8)	13	25 (5.2)	58 (12.5)	5 (2.7)
Skin. tissue and superficial vessel harm[1]	123 (5.9)	4	78 (8.1)	5	59 (12.4)	18 (4.5)	1 (1.1)	45 (4)	4	38 (8)	7 (1.5)	0 (0)
Peri-/post-operative bleeding/ haematoma - did not require re-operation	117 (5.6)	0	29 (3)	3	18 (3.8)	9 (2.3)	2 (2.2)	88 (7.8)	0	61 (12.8)	26 (5.6)	1 (0.5)
Pneumonia	117 (5.6)	4	77 (8)	3	47 (9.9)	25 (6.3)	5 (5.4)	40 (3.6)	5	21 (4.4)	13 (2.8)	6 (3.3)
Falls	87 (4.2)	15	38 (3.9)	19	6 (1.3)	22 (5.5)	10 (10.8)	49 (4.4)	10	17 (3.6)	24 (5.2)	8 (4.4)
Neurological[2]	87 (4.2)	2	46 (4.8)	2.5	33 (6.9)	11 (2.8)	2 (2.2)	41 (3.6)	2	30 (6.3)	9 (1.9)	2 (1.1)
Distended urinary bladder	81 (3.9)	3	52 (5.4)	3	39 (8.2)	12 (3)	1 (1.1)	29 (2.6)	3	24 (5)	5 (1.1)	0 (0)
Pulmonary embolism	64 (3.1)	27	15 (1.5)	12	3 (0.6)	8 (2)	4 (4.3)	49 (4.4)	32	9 (1.9)	15 (3.2)	25 (13.7)
Gastric ulcer	45 (2.2)	6	19 (2)	7	8 (1.7)	10 (2.5)	1 (1.1)	26 (2.3)	5.5	12 (2.5)	13 (2.8)	1 (0.5)
Cardiovascular[3]	40 (1.9)	3	14 (1.4)	13.5	6 (1.3)	5 (1.3)	3 (3.2)	26 (2.3)	3	17 (3.6)	7 (1.5)	2 (1.1)
Pain	36 (1.7)	4	7 (0.7)	3	4 (0.8)	3 (0.8)	0 (0)	29 (2.6)	9	13 (2.7)	13 (2.8)	3 (1.6)
Gastro-intestinal[4]	31 (1.5)	7	9 (0.9)	7	4 (0.8)	4 (1)	1 (1.1)	22 (2)	6.5	11 (2.3)	11 (2.4)	0 (0)
Peri-operative fracture	31 (1.5)	0	8 (0.8)	0	6 (1.3)	2 (0.5)	0 (0)	23 (2)	0	20 (4.2)	3 (0.6)	0 (0)
Renal failure	31 (1.5)	2	20 (2.1)	2	18 (3.8)	2 (0.5)	0 (0)	11 (1)	3	10 (2.1)	1 (0.2)	0 (0)
Deep vein thrombosis	29 (1.4)	42	10 (1)	24.5	0 (0)	6 (1.5)	4 (4.3)	19 (1.7)	46	0 (0)	6 (1.3)	13 (7.1)
Allergic reaction	25 (1.2)	5	8 (0.8)	5	4 (0.8)	4 (1)	0 (0)	17 (1.5)	4	11 (2.3)	4 (0.9)	2 (1.1)
Leg length difference	23 (1.1)	3	4 (0.4)	2.5	3 (0.6)	1 (0.3)	0 (0)	19 (1.7)	3	14 (2.9)	3 (0.6)	2 (1.1)

For peer review only

Gastrointestinal infection	22 (1.1)	9	17 (1.8)	11	8 (1.7)	8 (2)	1 (1.1)	5 (0.4)	4	4 (0.8)	1 (0.2)	0 (0)
Septicaemia	20 (1)	17.5	14 (1.4)	17.5	6 (1.3)	7 (1.8)	1 (1.1)	6 (0.5)	13.5	2 (0.4)	3 (0.6)	1 (0.5)
Mechanical complication[5]	20 (1)	25	7 (0.7)	24	3 (0.6)	4 (1)	0 (0)	13 (1.2)	29	2 (0.4)	5 (1.1)	6 (3.3)
Unclear infection	17 (0.8)	5	11 (1.1)	5	5 (1.1)	6 (1.5)	0 (0)	6 (0.5)	4	5 (1)	1 (0.2)	0 (0)
Respiratory	17 (0.8)	1	9 (0.9)	1	6 (1.3)	3 (0.8)	0 (0)	8 (0.7)	1	7 (1.5)	1 (0.2)	0 (0)
Myocardial infarction	15 (0.7)	7	6 (0.6)	3.5	3 (0.6)	3 (0.8)	0 (0)	9 (0.8)	8	5 (1)	3 (0.6)	1 (0.5)
Mouth and throat infection	12 (0.6)	9	8 (0.8)	9	5 (1.1)	3 (0.8)	0 (0)	4 (0.4)	10	2 (0.4)	2 (0.4)	0 (0)
Peripheral nerve injury[6]	12 (0.6)	2	3 (0.3)	8	1 (0.2)	2 (0.5)	0 (0)	9 (0.8)	2	8 (1.7)	1 (0.2)	0 (0)
Ileus	9 (0.4)	5	4 (0.4)	6	0 (0)	4 (1)	0 (0)	5 (0.4)	4	3 (0.6)	2 (0.4)	0 (0)
Stroke	9 (0.4)	4	4 (0.4)	5	3 (0.6)	1 (0.3)	0 (0)	5 (0.4)	4	3 (0.6)	1 (0.2)	1 (0.5)
Electrolyte imbalance	8 (0.4)	5.5	1 (0.1)	5	1 (0.2)	0 (0)	0 (0)	7 (0.6)	6	2 (0.4)	5 (1.1)	0 (0)
AE caused by anaesthesia[7]	7 (0.3)	1	3 (0.3)	0	3 (0.6)	0 (0)	0 (0)	4 (0.4)	1	3 (0.6)	1 (0.2)	0 (0)
Malnutrition	7 (0.3)	6	4 (0.4)	13	2 (0.4)	1 (0.3)	1 (1.1)	3 (0.3)	6	1 (0.2)	2 (0.4)	0 (0)
Bleeding - not related to surgery[8]	5 (0.2)	11	2 (0.2)	6.5	1 (0.2)	1 (0.3)	0 (0)	3 (0.3)	11	0 (0)	3 (0.6)	0 (0)
Infection other	5 (0.2)	11	4 (0.4)	11	1 (0.2)	3 (0.8)	0 (0)	1 (0.1)	6	0 (0)	1 (0.2)	0 (0)
Bleeding - that required re-operation	5 (0.2)	8	1 (0.1)	8	1 (0.2)	0 (0)	0 (0)	4 (0.4)	5	3 (0.6)	1 (0.2)	0 (0)
Surgical harm – other	3 (0.1)	71	0 (0)	NA	– (–)	– (–)	– (–)	3 (0.3)	71	0 (0)	0 (0)	3 (1.6)
Multi-organ failure	1 (0)	3	1 (0.1)	3	1 (0.2)	0 (0)	0 (0)	0 (0)	NA	– (–)	– (–)	– (–)
Other	10 (0.5)	7.5	3 (0.3)	19	1 (0.2)	1 (0.3)	1 (1.1)	7 (0.6)	7	2 (0.4)	4 (0.9)	1 (0.5)
Total	2093 (100)	8	968 (100)	8	476 (100)	399 (100)	93 (100)	1125 (100)	8	477 (100)	465 (100)	183 (100)

AE, adverse event.
Examples of adverse events in these types; [1] blister, extravasation, phlebitis; [2] acute confusion, hallucination, lethargy; [3] heart failure; [4] obstipation, vomiting, diarrhoea; [5] fracture without a fall, re-operation after several dislocations; [6] foot drop; [7] awareness, aspiration; [8] in connection to urinary catheter or warfarin treatment.

For peer review only

STROBE Statement—Checklist of items that should be included in reports of ***cohort studies***

	Item No	Recommendation	Page No
**Title and abstract**	1	(*a*) Indicate the study's design with a commonly used term in the title or the abstract	1
		(*b*) Provide in the abstract an informative and balanced summary of what was done and what was found	2
**Introduction**			
Background/rationale	2	Explain the scientific background and rationale for the investigation being reported	4
Objectives	3	State specific objectives, including any prespecified hypotheses	4
**Methods**			
Study design	4	Present key elements of study design early in the paper	5
Setting	5	Describe the setting, locations, and relevant dates, including periods of recruitment, exposure, follow-up, and data collection	5
Participants	6	(*a*) Give the eligibility criteria, and the sources and methods of selection of participants. Describe methods of follow-up	5
		(*b*) For matched studies, give matching criteria and number of exposed and unexposed	
Variables	7	Clearly define all outcomes, exposures, predictors, potential confounders, and effect modifiers. Give diagnostic criteria, if applicable	6
Data sources/ measurement	8*	For each variable of interest, give sources of data and details of methods of assessment (measurement). Describe comparability of assessment methods if there is more than one group	6-7
Bias	9	Describe any efforts to address potential sources of bias	NA
Study size	10	Explain how the study size was arrived at	5 (references)
Quantitative variables	11	Explain how quantitative variables were handled in the analyses. If applicable, describe which groupings were chosen and why	NA
Statistical methods	12	(*a*) Describe all statistical methods, including those used to control for confounding	7
		(*b*) Describe any methods used to examine subgroups and interactions	
		(*c*) Explain how missing data were addressed	
		(*d*) If applicable, explain how loss to follow-up was addressed	
		(*e*) Describe any sensitivity analyses	
**Results**			
Participants	13*	(a) Report numbers of individuals at each stage of study—eg numbers potentially eligible, examined for eligibility, confirmed eligible, included in the study, completing follow-up, and analysed	5
		(b) Give reasons for non-participation at each stage	
		(c) Consider use of a flow diagram	
Descriptive data	14*	(a) Give characteristics of study participants (eg demographic, clinical, social) and information on exposures and potential confounders	8-9
		(b) Indicate number of participants with missing data for each variable of interest	
		(c) Summarise follow-up time (eg, average and total amount)	
Outcome data	15*	Report numbers of outcome events or summary measures over time	8-13

For peer review only

Main results	16	(*a*) Give unadjusted estimates and, if applicable, confounder-adjusted estimates and their precision (eg, 95% confidence interval). Make clear which confounders were adjusted for and why they were included<br>(*b*) Report category boundaries when continuous variables were categorized<br>(*c*) If relevant, consider translating estimates of relative risk into absolute risk for a meaningful time period	NA
Other analyses	17	Report other analyses done—eg analyses of subgroups and interactions, and sensitivity analyses	NA
**Discussion**			
Key results	18	Summarise key results with reference to study objectives	14
Limitations	19	Discuss limitations of the study, taking into account sources of potential bias or imprecision. Discuss both direction and magnitude of any potential bias	17
Interpretation	20	Give a cautious overall interpretation of results considering objectives, limitations, multiplicity of analyses, results from similar studies, and other relevant evidence	14-17
Generalisability	21	Discuss the generalisability (external validity) of the study results	18
**Other information**			
Funding	22	Give the source of funding and the role of the funders for the present study and, if applicable, for the original study on which the present article is based	20

*Give information separately for exposed and unexposed groups.

Note: An Explanation and Elaboration article discusses each checklist item and gives methodological background and published examples of transparent reporting. The STROBE checklist is best used in conjunction with this article (freely available on the Web sites of PLoS Medicine at http://www.plosmedicine.org/, Annals of Internal Medicine at http://www.annals.org/, and Epidemiology at http://www.epidem.com/). Information on the STROBE Initiative is available at http://www.strobe-statement.org.

For peer review only

BMJ Open

Timing of adverse events in patients undergoing acute and elective hip arthroplasty surgery: a multi-centre cohort study using the Global Trigger Tool

Journal:	*BMJ Open*
Manuscript ID	bmjopen-2022-064794.R2
Article Type:	Original research
Date Submitted by the Author:	16-May-2023
Complete List of Authors:	Magnéli, Martin; Karolinska Institute, Department of Clinical Sciences at Danderyd Hospital; Danderyd University Hospital, Department of Orthopaedics<br>Kelly-Pettersson, Paula ; Karolinska Institutet Department of Clinical Sciences Danderyd Hospital, Orthopaedics; Danderyd University Hospital, Department of orthopaedics<br>Rogmark, Cecilia; Lund University, Department of Clinical Sciences Malmö, Clinical and Molecular Osteoporosis Research Unit; Skåne University Hospital Malmö Orthopedics Clinic, Department of Orthopaedics<br>Gordon, Max; Karolinska Institute, Department of clinical sciences at Danderyd hospital; Danderyd University Hospital, Department of orthopaedics<br>Sköldenberg, Olof; Karolinska Institutet, Department of clinical sciences at Danderyd Hospital; Danderyd University Hospital, Department of orthopaedics<br>Unbeck, Maria; Hogskolan Dalarna; Karolinska Institutet, Department of clinical sciences at Danderyd Hospital
**Primary Subject Heading**:	Surgery
Secondary Subject Heading:	Epidemiology
Keywords:	Orthopaedic & trauma surgery < SURGERY, Adult orthopaedics < ORTHOPAEDIC & TRAUMA SURGERY, Hip < ORTHOPAEDIC & TRAUMA SURGERY

SCHOLARONE™
Manuscripts

I, the Submitting Author has the right to grant and does grant on behalf of all authors of the Work (as defined in the below author licence), an exclusive licence and/or a non-exclusive licence for contributions from authors who are: i) UK Crown employees; ii) where BMJ has agreed a CC-BY licence shall apply, and/or iii) in accordance with the terms applicable for US Federal Government officers or employees acting as part of their official duties; on a worldwide, perpetual, irrevocable, royalty-free basis to BMJ Publishing Group Ltd ("BMJ") its licensees and where the relevant Journal is co-owned by BMJ to the co-owners of the Journal, to publish the Work in this journal and any other BMJ products and to exploit all rights, as set out in our licence.

The Submitting Author accepts and understands that any supply made under these terms is made by BMJ to the Submitting Author unless you are acting as an employee on behalf of your employer or a postgraduate student of an affiliated institution which is paying any applicable article publishing charge ("APC") for Open Access articles. Where the Submitting Author wishes to make the Work available on an Open Access basis (and intends to pay the relevant APC), the terms of reuse of such Open Access shall be governed by a Creative Commons licence – details of these licences and which Creative Commons licence will apply to this Work are set out in our licence referred to above.

Other than as permitted in any relevant BMJ Author's Self Archiving Policies, I confirm this Work has not been accepted for publication elsewhere, is not being considered for publication elsewhere and does not duplicate material already published. I confirm all authors consent to publication of this Work and authorise the granting of this licence.

For peer review only

Timing of adverse events in patients undergoing acute and elective hip arthroplasty surgery: a multi-centre cohort study using the Global Trigger Tool

Martin Magnéli[1,2*], Paula Kelly-Pettersson[1,2*], Cecilia Rogmark[3,4,5], Max Gordon[1,2], Olof Sköldenberg[1,2], Maria Unbeck[1,6]

[1] Karolinska Institutet, Department of Clinical Sciences, Danderyd Hospital, Unit of Orthopaedics, Stockholm, Sweden

[2] Danderyd University Hospital, Department of Orthopaedics, Stockholm, Sweden

[3] Skåne University Hospital, Department of Orthopaedics, Malmö, Sweden

[4] Lund University, Department of Clinical Sciences Malmö, Clinical and Molecular Osteoporosis Research Unit, Malmö, Sweden

[5] Swedish Arthroplasty Register, Centre of Registers Västra Götaland, Gothenburg, Sweden

[6] School of Health and Welfare, Dalarna University, Falun, Sweden

* Contributed equally

Corresponding author:
Martin Magnéli
Karolinska Institutet
Department of Clinical Sciences
Danderyd University Hospital
182 88 Stockholm
Sweden
martin.magneli@ki.se

Word count: 4235

For peer review only

ABSTRACT

Objective To explore timing in relation to all types of adverse events, severity and preventability for patients undergoing acute and elective hip arthroplasty.

Design A multi-centre cohort study using retrospective record review with Global Trigger Tool methodology in combination with data from several registers.

Setting 24 hospitals in 4 major regions of Sweden.

Participants Patients ≥18 years, undergoing acute or elective total or hemiarthroplasty of the hip were eligible for inclusion. Reviews of weighted samples of 1,998 randomly selected patient records were carried out using Global Trigger Tool methodology. The patients were followed for readmissions up to 90 days post-operatively throughout the whole country.

Results The cohort consisted of 667 acute and 1,331 elective patients. Most AEs occurred peri-and post-operatively (n=2093, 99.1%) and after discharge (n=1142, 54.1%). The median time from the day of surgery to the occurrence of AE was 8 days. The median days for different AE types ranged from 0 to 24.5 for acute and 0 to 71 for elective patients and peaked during different time periods. 40.2% of the AEs, both major and minor, occurred within post-operative day 0 to 5 and 86.9% of the AEs occurred within 30 days. Most of the AEs were deemed to be of major severity (n=1370, 65.5%) or preventable (n=1591, 76%).

Conclusions A wide variability was found regarding the timing of different adverse events with the majority occurring within 30 days. The timing and preventability varied regarding the severity. Most of the adverse events were deemed to be preventable and/or of major severity. To increase patient safety for patients undergoing hip arthroplasty surgery a better understanding of the multi-facetted nature of the timing of AEs in relation to the occurrence of differing AEs is needed.

For peer review only - http://bmjopen.bmj.com/site/about/guidelines.xhtml

Strengths and limitations of this study

- This is a registry-based national multicentre study including both acute and elective hip arthroplasty patients.
- Global Trigger Tool methodology for detecting adverse events was used for record review.
- All hospital admissions and unplanned outpatient visits occurring up to 90 days after surgery were reviewed.
- A weighted study sample optimised to select patients with adverse events was used to collect more data on adverse events compared with a random sample.

For peer review only

For peer review only - http://bmjopen.bmj.com/site/about/guidelines.xhtml

INTRODUCTION

Hip arthroplasty – "the operation of the century"[1] is an effective surgical treatment for both degenerative hip joint disease and femoral neck fractures. Still, adverse events (AEs) constituting a wide range of conditions can occur during the primary, index hospitalization and even years later. Both younger and older patients undergo hip arthroplasty nowadays, but the mean age of around 70 years indicates that a large group of elderly, and potentially frail patients undergo this surgery. This is particularly true for the group treated for femoral neck fractures.[2] AEs often entail suffering for the affected individual and the occurrence of preventable AEs reflects the gap between the actual care given and the expected standard of adequate safe care.[3 4]

Timing is an important factor in understanding the occurrence and prevention of AEs. It has been examined in some studies on arthroplasty surgery, but many of these included patients undergoing total primary hip or knee arthroplasty, and elective surgery was most common in these cohorts.[5-13] In contrast Bohl et al.[10] and Malik et al.[14], focused on geriatric patients undergoing hip arthroplasty after acute femoral neck fractures. Many of the timing studies[9-17] have used data from the American College of Surgeons National Surgical Quality Improvement Program with a follow-up period of 30 days post-operatively. This register does not include orthopaedic specific AEs such as dislocations.[9 12] Furthermore, many of these studies have had a rather narrow focus, for example, examining only peri-prosthetic joint infections[7], pulmonary embolism[8 18], venous thromboembolism[19], peri-prosthetic femur fractures[20], stroke[17 21], clostridium difficilie colitis[16], acute myocardial infarction[22 23] or a selection of pre-defined AEs.[5 9 10 12 14] In contrast, Parvizi et al.[6] and Yao et al.[11] have examined the occurrence of a broader range of AEs, but have not reported the timing for all the AEs that were identified.

There is a lack of knowledge regarding the timing of all types of AEs, both surgical and non-surgical, irrespective of whether they occur pre-, peri- or post-operatively in acute or elective procedures. . Furthermore, there is a need to examine timing in relation to the severity and preventability of AEs. Therefore, this study aimed to explore timing in relation to all types of adverse events, as well as severity and preventability in patients undergoing acute and elective hip arthroplasty.

For peer review only - http://bmjopen.bmj.com/site/about/guidelines.xhtml

METHODS

Study design

This sub-study is part of a retrospective multi-centre cohort study.[24] The aim of the main study was to validate the ability of a set of pre-defined ICD-10 codes used on a national level to compare hospitals following primary hip arthroplasties. The method and variables are the same for both the main study and the present study and are briefly described below. A more detailed description has been published previously.[24 25]

Participants and setting

The study population consisted of all patients aged ≥18 years undergoing either primary total hip arthroplasty or hemiarthroplasty, whether performed electively due to degenerative joint disease or acutely for a femoral neck fracture, that were entered into the SHAR during a three-year period (N=21,774). To increase the probability of selecting primary surgery admissions with the occurrence of at least one AE and to avoid excessive record review on admissions without AEs, we used a weighted sample. The study cohort was created by combining registry data from the Swedish Hip Arthroplasty Register (SHAR) and the National Patient Register. In total, 20 different selection groups for acute and elective arthroplasties were created.[24] The selection groups were based on the primary length of stay and readmission which were combined with patients that had pre-defined ICD-10 codes indicating AEs. Larger samples were drawn from the groups that had a high risk of AE (patients with extended length of stay, readmissions and AE ICD-10 codes).

The study cohort consisted of 2000 patients who had undergone either acute or elective primary surgery. The patients had their index surgery performed in hospitals located in one of four major regions in Sweden. Two patients were excluded leaving a final study cohort of 1998 patients. The patients were followed for a maximum of 90 days post-operatively for all in-patient and acute out-patient hospital care throughout the whole country, regardless of the location of the hospital where the index surgery was performed. Therefore, the study cohort had a total of 5423 admissions in 16 of the 21 regions in Sweden, with 69 hospitals involved, as individual patients may have had multiple admissions.

For peer review only - http://bmjopen.bmj.com/site/about/guidelines.xhtml

Data sources

This study used data from both medical records and three national registers, the National Patient Register, the Swedish Cause of Death Register, and the SHAR. In 2019, the SHAR had a completeness of 98% and 97% respectively for total hip replacements and hemiarthroplasties.[2] Data on primary surgery, such as date and type of surgery was obtained from the SHAR. This data was then cross-referenced with data from the National Patient Register using the Swedish personal identity number as a unique identifier. With this data, a timeline was created for each patient undergoing primary surgery and was used as a template to identify which admissions were to be reviewed.

We performed a manual retrospective record review using the Swedish version[26] of the Global Trigger Tool, a structured record review methodology.[27] This method involves a 2-stage review process.

Definitions

An AE was defined as suffering, physical harm or disease, as well as death related to the index admission and was not an inevitable consequence of the patient´s underlying disease or treatment.

A preventable adverse event was defined as an event that could have been prevented if adequate actions had been taken during the patient's contact with healthcare or social care.[28] A preventable event can be related to acts of commission or acts of omission.

The index admission was defined as the orthopaedic admission during which the patient had their primary hip arthroplasty surgery.

The occurrence date of the AE was defined as either the specific date when the sign or symptom first appeared or if this information was not available and the AE occurred after discharge the date when the patient contacted the care giver was then used. The occurrence date could also be the same date as the date of diagnosis.

Retrospective record review process

The record review process is described in detail elsewhere.[25] The recruitment of the ten reviewers (registered nurses, medical students, and physicians) was based on previous experience of record

For peer review only - http://bmjopen.bmj.com/site/about/guidelines.xhtml

review and/or convenience but all reviewers had previous experience of orthopaedic care.

A study manual, used as a complement to the Swedish Global Trigger Tool manual[26] with trigger definitions and descriptions, was created to clarify the study-specific interpretations and applications of triggers, definitions, and AE assessments. Triggers are clues or red flags in the medical record that indicate that a potential AE has occurred, for example, readmission within 30 days, re-operation.

The record review and time frame for the inclusion of an AE covered the period from the start of the admission to a maximum of 90 days post-operatively. During the follow-up period, it was possible to identify AEs in the records of patients receiving in-patient and/or out-patient care, irrespective of the speciality, at any hospital in Sweden.

The reviews were carried out in a 2-stage process using standardized data collection forms, one form for each review stage. In most cases, the same person carried out both stages of the review and the data was entered into a study-specific database.

In the first review stage, all record entries from all healthcare professionals were reviewed. The reviewers screened for the presence of one or more of the 38 pre-defined triggers in five modules of the Swedish version of the Global Trigger Tool.[26] Only records with triggers indicating at least one potential AE went forward to review stage 2.

In review stage 2, the reviewers sorted the different triggers into potential AEs, as more than one of the triggers can be involved in a single AE. Every potential AE was then reviewed separately. To qualify as an AE, a score of three or higher on a 4-point Likert scale was required (1=the AE was not related to index admission, 2=the AE was probably not related to index admission, 3=the AE was probably related to index admission, and 4=the AE was related to index admission). The preventability was also assessed using a similar scale with the same cut-off limit for inclusion regarding preventability.

Several other variables were collected, for example, the timing, type, and severity of the AEs. Severity was determined using a slightly modified version of the National Coordinating Council for Medication Error Reporting and Prevention (NCC MERP) Index.[29] NCC MERP Index categories E–I were included as they are related to harm i.e., AE. Events determined to be risks and no-harm incidents, i.e., NCC MERP categories A-D, were excluded.

For peer review only - http://bmjopen.bmj.com/site/about/guidelines.xhtml

Patient and public involvement

This study did not involve any patient or public representatives.

Statistical methods

Time from surgery to AE occurrence is presented in median days and interquartile range. The statistical analysis was performed using R 4.1.0 (R Project for Statistical Computing, Vienna, Austria), using tidyverse (v. 1.3.1) for dataset manipulation, ggplot2 (v. 3.3.5) for plots and htmlTable (v. 2.2.1) for creating tables.

RESULTS

Demographics

One third of the cohort were patients admitted acutely with a femoral neck fracture and two thirds underwent surgery on an elective basis. Patients with acute admissions were older, received hemiarthroplasties more often and were more often treated at university hospitals (table 1). Elective hemiarthroplasty represent a subgroup of patients, who all had the indication “Status post femoral neck fracture (FNF)” or avascular necrosis.

Table 1 Demographics

	**All patients n= 1998**	**Acute admitted n= 667**	**Elective admitted n= 1331**
Sex			
Female, n (%)	1250 (62.6)	444 (66.6)	806 (60.6)
Male, n (%)	748 (37.4)	223 (33.4)	525 (39.4)
Age, median years (min–max, IQR)	77 (18–100, 16)	84 (34–100, 10)	73 (18–99, 16)
LOS, median days (min–max, IQR)	6 (1–56, 5)	7 (1–56, 8)	5 (1–52, 3)
Type of surgery			
Total arthroplasty	1435 (71.8)	143 (21.4)	1292 (97.1)
Hemiarthroplasty	563 (28.2)	524 (78.6)	39 (2.9)
Type of hospital, n (%)			
University	630 (31.5)	295 (44.2)	335 (25.2)
Central County Council	556 (27.8)	180 (27.0)	376 (28.2)
County Council	531 (26.6)	109 (16.3)	422 (31.7)
Private*	281 (14.1)	83 (12.4)	198 (14.9)

LOS, length of stay, IQR, interquartile range.
* Most of the surgeries are publicly financed through agreements with the regional authorities.
Note: Weighted samples, the values are not representative for average Swedish orthopaedic care concerning hip arthroplasty.

For peer review only

Adverse event outcomes

In total, 2116 AEs of varying severity were identified in 1171 (58.6%) patients in our weighted sample of which 527 (45.0%) of these patients were affected by more than one AE. The patients admitted acutely sustained 981 (46.4%; min to max, 1–10) AEs and the elective patients 1135 (53.6%; min to max 1–7). Acute patients were affected by an AE to a higher extent compared to elective patients, 71.4% (476/667) versus 52.3% (696/1331). The patients in the cohort had in total 3425 all-cause re-admissions during the follow-up period. However, some of the re-admissions were not related to the index admission and were therefore not considered to be related to an AE.

Timing overall and in connection to adverse event types

Four AEs did not have a correct date registered and were excluded, leaving 2112 AEs remaining for the timing analysis, 980 AEs in acute patients and 1132 AEs in elective patients. Nineteen (0.9%) AEs occurred from admission to the day before surgery and 2093 (99.1%) AEs occurred from the day of surgery, including peri-operative AEs, up to 90 days post-operatively.

Of the 2112 AEs, 970 (45.9%) occurred during the index orthopaedic admission, the remaining 1142 (54.1%) AEs occurred after discharge. The AEs occurring after discharged were distributed as follow: 866 (41.0%) within 30 days and 276 (13.1%) between 31 and 90 days after surgery. Of the patients undergoing acute surgery 486 (49.6%) of the 980 AEs occurred during the index admission, compared to elective patients where 484 (42.8%) of 1132 AEs occurred during the index admission.

The median time from day of surgery to an AE was 8 days for the 2093 AEs occurring on the day of surgery and post-operatively. The corresponding median for both acute and elective patients was also 8 days (summary in table 2, full table in supplementary material).

For both acute and elective patients, pressure ulcers, skin, tissue and superficial vessel harm, peri- and post-operative bleeding/haematomas not requiring re-operation and pneumonia were common AEs and peaked during the index admission. Within 30 days after surgery, but after discharge, dislocation of the prothesis and infections such as deep peri-prosthetic infections, superficial wound infections and urinary tract infections were commonly occurring AEs and peaked in this period for both groups. No AE peaked after 30 days post-operatively for the acute patients, whereas for the elective patients, pulmonary embolisms, deep venous thromboses, mechanical

For peer review only - http://bmjopen.bmj.com/site/about/guidelines.xhtml

complications and surgical harm – other all peaked after 30 days. Some types of AEs continued to occur at a high rate such as dislocations in both groups after 30 days (table 2).

For peer review only

For peer review only - http://bmjopen.bmj.com/site/about/guidelines.xhtml

**Table 2** Summary of identified adverse events for acute respective elective patients, sorted by descending order of types of AEs for all patients. Full table available in supplementary material.						
	**All**		**Acute**		**Elective**	
**Type of AEs**	**N (%)**	**Median time from surgery to AE (days)**	**n (%)**	**Median time from surgery to AE (days)**	**n (%)**	**Median time from surgery to AE (days)**
Dislocation of the prothesis	274 (13.1)	23	101 (10.4)	23	173 (15.4)	23
Pressure ulcer	189 (9)	5	143 (14.8)	5	46 (4.1)	4.5
Urinary tract infection	163 (7.8)	8	93 (9.6)	9	70 (6.2)	8
Peri-prosthetic joint infection	149 (7.1)	20	36 (3.7)	19	113 (10)	20
Superficial wound infection	147 (7)	11	59 (6.1)	11	88 (7.8)	13
Skin. tissue and superficial vessel harm[1]	123 (5.9)	4	78 (8.1)	5	45 (4)	4
Peri-/post-operative bleeding/ haematoma - did not require re-operation	117 (5.6)	0	29 (3)	3	88 (7.8)	0
Pneumonia	117 (5.6)	4	77 (8)	3	40 (3.6)	5
Falls	87 (4.2)	15	38 (3.9)	19	49 (4.4)	10
Neurological[2]	87 (4.2)	2	46 (4.8)	2.5	41 (3.6)	2
Distended urinary bladder	81 (3.9)	3	52 (5.4)	3	29 (2.6)	3
Pulmonary embolism	64 (3.1)	27	15 (1.5)	12	49 (4.4)	32
Gastric ulcer	45 (2.2)	6	19 (2)	7	26 (2.3)	5.5
Cardiovascular[3]	40 (1.9)	3	14 (1.4)	13.5	26 (2.3)	3
Pain	36 (1.7)	4	7 (0.7)	3	29 (2.6)	9
Gastro-intestinal[4]	31 (1.5)	7	9 (0.9)	7	22 (2)	6.5
Peri-operative fracture	31 (1.5)	0	8 (0.8)	0	23 (2)	0
Renal failure	31 (1.5)	2	20 (2.1)	2	11 (1)	3
Deep vein thrombosis	29 (1.4)	42	10 (1)	24.5	19 (1.7)	46
Allergic reaction	25 (1.2)	5	8 (0.8)	5	17 (1.5)	4
Leg length difference	23 (1.1)	3	4 (0.4)	2.5	19 (1.7)	3
Gastrointestinal infection	22 (1.1)	9	17 (1.8)	11	5 (0.4)	4
Septicaemia	20 (1)	17.5	14 (1.4)	17.5	6 (0.5)	13.5
Mechanical complication[5]	20 (1)	25	7 (0.7)	24	13 (1.2)	29
Unclear infection	17 (0.8)	5	11 (1.1)	5	6 (0.5)	4
Respiratory	17 (0.8)	1	9 (0.9)	1	8 (0.7)	1
Myocardial infarction	15 (0.7)	7	6 (0.6)	3.5	9 (0.8)	8
Mouth and throat infection	12 (0.6)	9	8 (0.8)	9	4 (0.4)	10
Peripheral nerve injury[6]	12 (0.6)	2	3 (0.3)	8	9 (0.8)	2
Ileus	9 (0.4)	5	4 (0.4)	6	5 (0.4)	4
Stroke	9 (0.4)	4	4 (0.4)	5	5 (0.4)	4
Electrolyte imbalance	8 (0.4)	5.5	1 (0.1)	5	7 (0.6)	6
AE caused by anaesthesia[7]	7 (0.3)	1	3 (0.3)	0	4 (0.4)	1
Malnutrition	7 (0.3)	6	4 (0.4)	13	3 (0.3)	6
Bleeding - not related to surgery[8]	5 (0.2)	11	2 (0.2)	6.5	3 (0.3)	11
Infection other	5 (0.2)	11	4 (0.4)	11	1 (0.1)	6
Bleeding - that required re-operation	5 (0.2)	8	1 (0.1)	8	4 (0.4)	5

For peer review only - http://bmjopen.bmj.com/site/about/guidelines.xhtml

Surgical harm – other	3 (0.1)	71	0 (0)	NA	3 (0.3)	71
Multi-organ failure	1 (0)	3	1 (0.1)	3	0 (0)	NA
Other	10 (0.5)	7.5	3 (0.3)	19	7 (0.6)	7
Total	2093 (100)	8	968 (100)	8	1125 (100)	8

AE, adverse event.
Examples of adverse events in these types; [1] blister, extravasation, phlebitis; [2] acute confusion, hallucination, lethargy; [3] heart failure; [4] obstipation, vomiting, diarrhoea; [5] fracture without a fall, re-operation after several dislocations; [6] foot drop; [7] awareness, aspiration; [8] in connection to urinary catheter or warfarin treatment.

The 8 most common types of AEs and their median day of occurrence are shown in figure 1. Of these, peri-/post-operative bleeding/haematoma had the earliest day of occurrence at 3 (interquartile range, 12) days for acute patients and 0 (7) days for elective patients. The second earliest occurring AE was skin, tissue and superficial vessel harm with a median of 5 (9) for acute and 4 (4) days for the elective patients. Dislocations had the longest median time to occurrence with 23 (24 versus 29) days for both groups.

Add figure 1 here.

Timing outcomes within 5 days post-operatively

In total, 40.2% of both minor and major AEs (n=842/2093) occurred either on the day of surgery or during the following 5 post-operative days (table 3). Some types of AEs were only common early in the 5-day time frame, for example, peri-operative haemorrhage, peri-operative fractures, and dislocation on post-operative day 1 while some other AEs such as skin, tissue and superficial vessel harm and distended urinary bladder were frequent from the day of surgery and onwards. Later in the time frame, infections such as pneumonia, urinary tract infections and superficial wound infections began to occur more commonly.

Table 3 Peri-operative and early post-operative adverse events (AEs), the 5 most common AE types per day

Type of AE, n	POD 0	POD 1	POD 2	POD 3	POD 4	POD 5
Peri-operative haemorrhage	59					
Peri-operative fracture	25					
Skin, tissue and superficial vessel harm	11	14		17	10	11
Neurological	9	20	18			
Distended urinary bladder	8	9	16	17	8	
Dislocation		14				
Pressure ulcer		14	21	30	17	15
Pneumonia			27	18		7
Urinary tract infection			14	11	13	5
Superficial wound infection					12	5

For peer review only - http://bmjopen.bmj.com/site/about/guidelines.xhtml

Total for the 5 most common per day, (%) of total per day	112 (64.0)	71 (48.6)	96 (56.8)	93 (58.5)	60 (55.0)	43 (51.2)
Total for all AEs per day	175	146	169	159	109	84
POD, post-operative day.						

Timing and preventability in relation to severity

Preventability and timing varied within and between the acute and elective groups, as well as, for the different AE types regarding severity. Most of the AEs were deemed to be of major severity (n=1370, 65.5%, NCC MERP categories F-I) or preventable (n=1591, 76.0%). The proportion of major AEs was higher among the elective patients compared to the acute, 72.9% versus 56.8%. In contrast, the proportion of AEs that contributed to death was higher among the acute patients, 3.9% versus 0.5%. All deaths in the elective group were deemed preventable and the AEs contributing to death began to occur later (median days 20.5) in contrast to the 65.9% preventable AEs for the acute group with a median of 15 days (table 4). Median days to death was in total 29 days for 28 acute patients and 25 days for 4 elective patients. Examples of AEs resulting in some degree of permanent harm, classified according to the Swedish patient insurance assessment standards, were dislocations, deep peri-prosthetic infections, fractures, thromboses/embolisms, and leg length difference.

In total, a third of the AEs were classified as minor (NCC MERP category E) including, for example, pressure ulcers, urinary tract infections, skin harm, falls with minor injury, distended urinary bladders, and intravenous infiltrations. In total, minor AEs and AEs that required intervention necessary to sustain life within 60 minutes (NCC MERP category H) occurred earlier (median 4 respective 1 day) compared to the other NCC MERP categories. Among the acute patients, minor AEs occurred more often, although somewhat later and were assessed to be more preventable compared those in elective patients (table 4).

Table 4 Number, proportion, and timing of adverse events (AEs) and preventable AEs in relation to severity

	**Severity according to NCC MERP**					
Groups	E	F	G	H	I	Total
**Adverse events, all patients**						
N (%) of AEs	723 (34.5)	794 (37.9)	518 (24.7)	14 (0.7)	44 (2.1)	2093 (100)
Median (IQR) days	4 (7)	8 (15)	21 (27)	1 (3)	15.5 (18.75)	8 (16)
N (%) of preventable AEs	528 (73.0)	541 (68.1)	482 (93.0)	11 (78.6)	29 (65.9)	1,591 (76.0)
Median (IQR), days, preventable AEs	4 (7)	8 (14)	21 (27)	1 (2.5)	16 (18)	9 (17)
**Adverse events, acute patients**						
N (%) of AEs	418 (43.2)	335 (34.6)	169 (17.5)	8 (0.8)	38 (3.9)	968 (100)
Median (IQR) days	5 (8)	7 (13)	22 (23)	1 (1.75)	15 (20.25)	8 (14)
N (%) of preventable AEs	321 (76.8)	243 (72.5)	155 (91.7)	8 (100)	23 (60.5)	750 (77.5)
Median (IQR), days, preventable AEs	5 (7)	7 (12.5)	22 (20.5)	1 (1.75)	15 (19.5)	8 (14.75)
**Adverse events, elective patients**						
N (%) of AEs	305 (27.1)	459 (40.8)	349 (31.0)	6 (0.5)	6 (0.5)	1125 (100)

For peer review only - http://bmjopen.bmj.com/site/about/guidelines.xhtml

Median (IQR) days	3 (6)	8 (16)	21 (31)	1.5 (6.25)	20.5 (13)	8 (18)
N (%) of preventable AEs	207 (67.9)	298 (64.9)	327 (93.7)	3 (50.0)	6 (100)	841 (74.8)
Median (IQR), days, preventable AEs	3 (5)	8.5 (15.75)	21 (29)	1 (14.5)	20.5 (13)	10 (19)

NCC MERP, National Coordinating Council for Medication Error Reporting and Prevention Index.
E, Contributed to or resulted in temporary harm; F, Contributed to or resulted in temporary harm that prolonged hospitalization, required out-patient care or readmission; G, Contributed to or resulted in permanent patient harm; H, Required intervention necessary to sustain life within 60 minutes; I, Contributed or resulted in the patient's death
IQR, interquartile range.

For peer review only

For peer review only - http://bmjopen.bmj.com/site/about/guidelines.xhtml

DISCUSSION

To the best of our knowledge, this is the first study to explore all types of AEs with associated timing data, both in total and separately for acute and elective patients. Most of the AEs occurred after the index admission in both groups. As much as 87% of the AEs occurred within 30 days post-operatively. The overall median time of 8 days from the day of surgery to an AE was the same for both acute and elective patients. Pressure ulcers and skin, tissue and superficial vessel harm were common and peaked for both group during the index admission. Dislocations and deep peri-prosthetic joint infections were common reasons for readmissions within 30 days after surgery and peaked during this time frame. Dislocations continued to occur to a high extent after 30 days. Forty percent of all AEs, both major and minor, occurred from the day of surgery and up to and including the fifth post-operative day. The timing and preventability varied regarding the severity. Most of the AEs were deemed to be preventable and/or of major severity.

Previous timing studies examining patients undergoing arthroplasty have used varying data collection methods, included varying types of arthroplasty surgery, often with a focus on total hip replacements in elective patients, and the criteria for AEs, timing and follow-up periods differ. Furthermore, some studies do not report data for hip arthroplasty separately. This makes comparison of the outcomes somewhat difficult.

Type of adverse events and timing in general

A wide variability was found regarding the timing of AEs. Peri-/post-operative bleeding/ haematoma, peri-operative fracture, AEs caused by anaesthesia, respiratory AEs, renal failure, peripheral nerve injuries and neurological AEs occurred early, within the first 2 days post-operatively. The latest occurring AEs included surgical harms of other types, deep vein thromboses, mechanical complications, dislocations and peri-prosthetic infections which ranged from median 20 to 71 days. Some AEs continued to occur up until the end of the follow-up period.

Dislocation was the most common AE in elective patients and the second most common in acute patients. It occurred at a median of 3 weeks and in 9 of 10 cases after discharge. This AE is seldom reported in timing studies, for example, the commonly used National Surgical Quality Improvement Program does not include dislocations and other orthopaedic-specific AEs.[9 12] However, Ali et al.[30] also found that dislocation was one of the most common AEs which clustered early after discharge. In their study, after 30 days post-operatively 74% of the

For peer review only - http://bmjopen.bmj.com/site/about/guidelines.xhtml

dislocations requiring readmission had occurred and this was higher than the 51% in the present study. Even after 30 days, dislocations continued to occur at a high rate.

Other common AEs were infections such as peri-prosthetic, urinary tract and superficial wound infections, the majority of which occurred after discharge. The median time to superficial wound infections and peri-prosthetic infections was 11 versus 20 days and the corresponding for Malik et al.[14] was 16 versus 23 days. Bohl et al.[9 10] reported in two studies surgical site infections with median days of 17 and 16, respectively. Our median time to urinary tract infections is in line with results from other studies with a range of 7 to 8 days.[9 10 14]

In contrast to other AEs, pulmonary embolism and deep vein thrombosis occurred relatively late at 4 and 6 weeks respectively. The longer time to the occurrence of deep vein thrombosis compared to pulmonary embolism can also be seen in other studies.[5 9 10 14] In contrast, these two AE types occurred later in our cohort than in other studies where median days to pulmonary embolism and deep vein thrombosis ranged from 3-5 days and 6-9 days, respectively.[5 9 10 14] A partial explanation can be that we had a longer follow-up period which affects the median day outcomes.

Several of our most common AE types, such as, pressure ulcers, skin, tissue and superficial vessel harm, falls, neurological harm, and distended urinary bladder are not covered by similar timing studies examining AEs in hip arthroplasty patients. These AEs, which mostly peaked during the index admission, were often classified as minor and preventable but not directly related to the surgical intervention. Nevertheless, minor AEs may also lead to suffering for the patient. To reduce the number of AEs occurring in orthopaedic care, it is important to analyse AEs also from a timing perspective and work proactively pre-, peri- and post-operatively in interprofessional teams both inside and outside the operating room.[31]

Two timing studies focused on geriatric patients with hip fractures in general[14] and those treated with arthroplasty.[10] Although, timing of specific AEs was quite similar, pulmonary embolism, deep vein thrombosis and sepsis occurred somewhat later and superficial wound infections occurred somewhat earlier in our acute cohort. The patterns of AE types in relation to early or late onset were similar for the most part.

Timing during and after index admission, early and late onset

We found that less than half of all AEs occurred during the index admission. The median length of index stay is longer in our study compared to several other studies. This can be explained by

For peer review only - http://bmjopen.bmj.com/site/about/guidelines.xhtml

the fact that we included acute patients and admissions with longer length of stay were over-represented due to the sampling technique used in the main study. Nevertheless, other studies with shorter length of stay found that most of the AEs occurred during the index admission.[9 10 12] In contrast, Yao et al.[11] had a higher AE occurrence after discharge when studying a cohort of home-admitted patients. As we are moving toward shorter hospital stays and an increased amount of day-surgery, the risk of major AEs occurring outside the hospitals increases. Therefore, a careful follow-up plan after discharge can be of value. Co-production of healthcare outcomes by the healthcare professionals and the patient as an important participating partner may be a significant intervention in the preventive work to reduce the occurrence of AEs and increase patient safety.[32] The patients, and in some cases their significant others and the staff at nursing homes, need to be well informed regarding which signs and symptoms of AEs to be aware of and how to manage self-care. These are central aspects during the rehabilitation period.

Most of the AEs in the present study occurred within 30 days post-operatively. Many other studies had a follow-up period of only 30 days and most of the AEs would probably be detected during this period. However, this time frame may lead to a risk of underestimation of the incidence of AEs and a bias towards the earlier-diagnosed AEs as some AE types, for example, surgical site infections did not plateau within this specific follow-up period.[9 10 14]

Nearly half of all AEs occurred within the first 5 days post-operatively. Still, none of the 4 "catastrophic" AEs, pre-defined by Johnson et al.[12] namely pulmonary embolism, myocardial infarction or cardiac arrest, cerebrovascular accident, or death were among our 5 most common AEs during this time period. The corresponding results for Parvizi et al.[6] was 93% for life-threating medical AEs. Belmont et al.´s[13] findings showed that 77% of total hip arthroplasty patients who had an adverse cardiac event in the form of myocardial infarction or cardiac arrest experienced it within 3 days after surgery.

Severity and preventability

The timing and preventability varied regarding the severity for the acute and elective patients. We assessed severity with a widely used scale in AE studies using the Global Trigger Tool record review methodology.[27] The severity of each AE was assessed independently, and a specific type of AE, for example pressure ulcers, falls and urinary tract infections could be assessed as having varying degrees of severity depending on the harm caused. Parvizi et al.[6] and Yao et al.[11] classified all, for example, pneumonia as minor AEs while we classified them

For peer review only - http://bmjopen.bmj.com/site/about/guidelines.xhtml

as major. On the other hand, Parvizi et al.[6] defined AEs that resulted in, for example, prolonged hospital stays as minor while we classified these AEs as major (NCC MERP F).

In contrast to other studies[5 6 11-14], we did not include death as a separate AE type as we considered death to be the consequence of an AE and belongs to the severity classification. The median time to death was around 4 weeks for both groups. For some patients more than one AE may have contributed to their death (NCC MERP category I).

Further research is needed to explore the multi-facetted nature of the timing in relation to the occurrence of minor and major AEs, both surgical and non-surgical, during the pre-, peri- or post-operative periods of patient care. A multi-pronged approach is required in the prevention of AEs[14], as most AEs were deemed to be preventable, and patients were commonly affected by more than one AE. To increase patient safety for this patient group, additional research is needed regarding the implementation and evaluation of timely targeted interventions during the varying phases of the patient-care process, focusing on preventable AEs.

Limitations and strengths

The study has several limitations. We have not included AEs occurring after 90 days post-operatively. Using record review as the data collection method may have led to an underestimation of AEs due to underreporting. Furthermore, AEs detected and treated outside the hospital setting may have been missed. These are probably minor AEs as severe AEs would be more likely to be treated in a hospital setting. This has led to severe outcome, for example, peri-prosthetic joint infections being more common compared to superficial wound infections. Even though we reported the time to occurrence in most cases, some AEs occurring after discharge may have had a delayed timing date if the patient was not specific enough about when the first symptom of the AE occurred. In these cases, the date when the patient first contacted the care giver was used. The weighted study sample used in this study was useful for maximising the inclusion of patients with an AE in relation to the main study´s aim and this is the explanation for the high AE rates. This is not the incidence of AEs in the source population. The estimated incidence for different AEs has been calculated by adjusting for the sampling weights in previous publications from this study.[24]

The multi-centre design with a wide range of patients of all ages and types of hospitals can be considered as a strength. The 90-day national follow-up, regardless of index hospital should be sufficient to detect most acute, sub-acute and rare AEs. To ensure the reliability and validity of

For peer review only - http://bmjopen.bmj.com/site/about/guidelines.xhtml

our data, the study has been closely monitored to check the correctness and completeness of the data, enabling for a good control of the review process. We used a stringent definition of what constitutes an AE and attained very good kappa values in the review process[24]. We only included AEs related to the care given and excluded conditions judged to be related to an underlying disease or condition, unless the disease or condition deteriorated due to healthcare. We believe our results to be valid and generalizable at least to Western world patients and/or publicly financed healthcare.

For peer review only

For peer review only - http://bmjopen.bmj.com/site/about/guidelines.xhtml

Acknowledgements

The authors wish to thank Marie Ax, Susanne Hansson, Zara Hedlund, Mirta Stupin, Tim Hansson, Lovisa Hult-Ericson, and Christina Jansson for their help and support with the review records. We would also like to thank all departmental managers for providing access to the patient records and Per Nydert for his help with the study database.

Contributors

MM collected data, analysed the data, and critically revised the manuscript.
PKP interpreted the data and contributed to the drafting of the work.
CR contributed to the design of the study and contributed to the drafting of the work.
MG contributed to the design of the study, collected data, and critically revised the manuscript.
OS contributed to the design of the study, collected data, and critically revised the manuscript.
MU contributed to the design of the study, monitored the reviews, analysed and interpreted the data, and was mainly responsible for the drafting of the work.
All authors have approved the final version of the manuscript and agree to be accountable for all aspects of the work.

Competing interests

None to declare.

Data sharing statement

No additional data is available.

Ethical approval

Ethical approval was provided by the Regional Ethics Committee of Gothenburg (516-13 and T732-13). Permission for data access for the reviewers was granted by the head of each respective unit. The patients did not provide an informed consent to the record review.

For peer review only

Funding

This work was supported by the Swedish Patient Insurance. The funder has not been involved in any part of the study, in either writing the manuscript or the decision to submit the manuscript for publication.

Patient and Public involvement

Patients and/or the public were not involved in the design, conduct, reporting or dissemination plans of our research.

Patient consent for publication

Not applicable.

ORCHID IDs

Martin Magnéli https://orchid.org/ 0000-0003-0341-0227

Paula Kelly-Pettersson https://orchid.org/0000-0003-3685-2953

Cecilia Rogmark https://orchid.org/0000-0002-6556-4320

Max Gordon https://orchid.org/ 0000-0002-8080-5815

Olof Sköldenberg https://orchid.org/ 0000-0003-4155-8425

Maria Unbeck https://orchid.org/ 0000-0002-5090-0352

For peer review only

REFERENCES

1 Learmonth ID, Young C, Rorabeck C. The operation of the century: total hip replacement. *Lancet* 2007;370:1508-19.

2 W-Dahl A, Kärrholm J, Nauclér E, *et al*. Annual report 2021. The Swedish Arthroplasty Register. Available: https://registercentrum.blob.core.windows.net/slr/r/SAR-Annual-Report-2021-SJlAFmlRl5.pdf [Accessed 10 May 2022].

3 Duarte Sda C, Stipp MA, da Silva MM, *et al*. Adverse events and safety in nursing care. *Rev Bras Enferm* 2015;68:136-46, 144-54.

4 Pettersson PK, Skoldenberg O, Samuelsson B, *et al*. The identification of adverse events in hip fracture patients using the Global Trigger Tool: A prospective observational cohort study. *Int J Orthop Trauma Nurs* 2020;38:100779.

5 Mantilla CB, Horlocker TT, Schroeder DR, *et al*. Frequency of myocardial infarction, pulmonary embolism, deep venous thrombosis, and death following primary hip or knee arthroplasty. *Anesthesiology* 2002;96:1140-6.

6 Parvizi J, Mui A, Purtill JJ, *et al*. Total joint arthroplasty: When do fatal or near-fatal complications occur? *J Bone Joint Surg Am* 2007;89a:27-32.

7 Pulido L, Ghanem E, Joshi A, *et al*. Periprosthetic joint infection: The incidence, timing, and predisposing factors. *Clin Ortho Relat Res* 2008;466:1710-15.

8 Parvizi J, Huang R, Raphael IJ, et al. Timing of symptomatic pulmonary embolism with warfarin following arthroplasty. *J Arthroplasty* 2015;30:1050-3.

9 Bohl DD, Ondeck NT, Basques BA, *et al*. What is the timing of general health adverse events that occur after total joint arthroplasty? *Clin Orthop Relat Res* 2017;475:2952-59.

10 Bohl DD, Samuel AM, Webb ML, *et al*. Timing of adverse events following geriatric hip fracture surgery: A Study of 19,873 patients in the American college of surgeons national surgical quality improvement program. *Am J Orthop (Belle Mead NJ)* 2018;47.

11 Yao DH, Keswani A, Shah CK, *et al*. Home discharge after primary elective total joint arthroplasty: postdischarge complication timing and risk factor analysis. *J Arthroplasty* 2017;32:375-80.

12 Johnson DJ, Hartwell MJ, Weiner JA, *et al*. Which postoperative day after total joint arthroplasty are catastrophic events most likely to occur? *J Arthroplasty* 2019;34:2466-72.

13 Belmont PJ, Goodman GP, Kusnezov NA, *et al*. Postoperative myocardial infarction and cardiac arrest following primary total knee and hip arthroplasty: rates, risk factors, and time of occurrence. *J Bone Joint Surg Am* 2014;96a:2025-31.

For peer review only - http://bmjopen.bmj.com/site/about/guidelines.xhtml

14 Malik AT, Quatman CE, Phieffer LS, *et al*. Timing of complications following surgery for geriatric hip fractures. *J Clin Orthop Trauma* 2019;10:904-11.

15 Minhas SV, Kester BS, Lovecchio FC, et al. Nationwide 30-day readmissions after elective orthopedic surgery: reasons and implications. *J Healthc Qual* 2017;39:34-42.

16 Bovonratwet P, Bohl DD, Malpani R, *et al*. Incidence, risk factors, and impact of clostridium difficile colitis following primary total hip and knee arthroplasty. *J Arthroplasty* 2018;33:205-10.e1.

17 Haynes MS, Alder KD, Bellamkonda K, *et al*. Incidence, predictors, and timing of post-operative stroke following elective total hip arthroplasty and total knee arthroplasty. *PLoS One* 2020;15:e0239239.

18 Hu C, Liu C, Wang Y, *et al*. The timing of symptomatic pulmonary embolism in patients with nonwarfarin anticoagulants following elective primary total joint arthroplasty. *J Arthroplasty* 2020;35:1703-07.

19 Shohat N, Goel R, Ludwick L, *et al*. Time to venous thromboembolism events following total hip arthroplasty: A comparison between aspirin and warfarin. *J Arthroplasty* 2022. [Published Online First: 2022/02/13]

20 Lygrisse KA, Gaukhman GD, Teo G, et al. Is surgical approach for primary total hip arthroplasty associated with timing, incidence, and characteristics of periprosthetic femur fractures? *J Arthroplasty* 2021;36:3305-11.

21 Lalmohamed A, Vestergaard P, Cooper C, *et al*. Timing of stroke in patients undergoing total hip replacement and matched controls: a nationwide cohort study. *Stroke* 2012;43:3225-9.

22 Lalmohamed A, Vestergaard P, Klop C, *et al*. Timing of acute myocardial infarction in patients undergoing total hip or knee replacement: a nationwide cohort study. *Arch Intern Med* 2012;172:1229-35.

23 Gandhi R, Petruccelli D, Devereaux PJ, et al. Incidence and timing of myocardial infarction after total joint arthroplasty. *J Arthroplasty* 2006;21:874-7.

24 Magnéli M, Unbeck M, Rogmark C, et al. Validation of adverse events after hip arthroplasty: a Swedish multi-centre cohort study. *BMJ Open* 2019;9:e023773.

25 Hommel A, Magneli M, Samuelsson B, et al. Exploring the incidence and nature of nursing-sensitive orthopaedic adverse events: A multicenter cohort study using Global Trigger Tool. *Int J Nurs Stud* 2020;102:103473.

For peer review only - http://bmjopen.bmj.com/site/about/guidelines.xhtml

26 SALAR. *Marker-based record review: to identify and measure harm in healthcare* [in Swedish: Markörbaserad journalgranskning: för att identifiera och mäta skador i vården]. Stockholm: Swedish Association of Local Authorities and Regions (SALAR), 2012.

27 Griffin FA, Resar, R.K. *IHI Global Trigger Tool for measuring adverse events* (second edition). IHI Innovation Series white paper. Cambridge, MA: Institute for Healthcare Improvement, 2009.

28 Swedish Codes of Statutes. Patient Safety Act (2010:659) [In Swedish: Patientsäkerhetslag 2010:659]. Available: https://www.riksdagen.se/sv/dokument-lagar/dokument/svensk-forfattningssamling/patientsakerhetslag-2010659_sfs-2010-659 [Accessed 10 May 2022].

29 National Coordinating Council for Medication Error Reporting and Prevention. Available: https://www.nccmerp.org/types-medication-errors [Accessed 10 May 2022].

30 Ali AM, Loeffler MD, Aylin P, *et al*. Timing of readmissions after elective total hip and knee arthroplasty: Does a 30-day all-cause rate capture surgically relevant readmissions? *J Arthroplasty* 2021;36:728-33.

31 Unbeck M, Dalen N, Muren O, *et al*. Healthcare processes must be improved to reduce the occurrence of orthopaedic adverse events. *Scand J Caring Sci* 2010;24:671-7.

32 World Health Organization. Exploring patient participartion in reducing health-care-related safety risks. 2013. Available: https://www.euro.who.int/__data/assets/pdf_file/0010/185779/e96814.pdf [Accessed 29 April 2022].

For peer review only - http://bmjopen.bmj.com/site/about/guidelines.xhtml

Legends

Figure 1. Median days from the day of surgery for the 8 most common types of adverse events up to 90 days post-operatively.

For peer review only

For peer review only - http://bmjopen.bmj.com/site/about/guidelines.xhtml

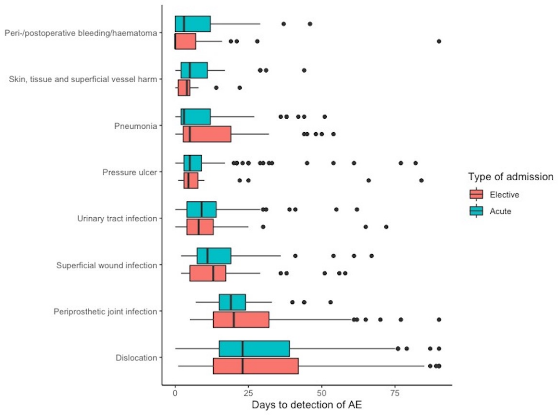

For peer review only

Table 2 Overview of identified adverse events for acute respective elective patients during different time frames and time to occurrence, sorted by descending order of types of AEs for all patients

	All		Acute					Elective				
**Type of AEs**	**N (%)**	**Median time from surgery to AE (days)**	**n (%)**	**Median time from surgery to AE (days)**	**Occurred during index admission n (%)**	**Occurred within 30 days but after index n (%)**	**Occurred within 31–90 days n (%)**	**n (%)**	**Median time from surgery to AE (days)**	**Occurred during index admission n (%)**	**Occurred within 30 days but after index n (%)**	**Occurred within 31–90 days n (%)**
Dislocation of the prothesis	274 (13.1)	23	101 (10.4)	23	11 (2.3)	56 (14)	34 (36.6)	173 (15.4)	23	23 (4.8)	83 (17.8)	67 (36.6)
Pressure ulcer	189 (9)	5	143 (14.8)	5	91 (19.1)	46 (11.5)	6 (6.5)	46 (4.1)	4.5	33 (6.9)	11 (2.4)	2 (1.1)
Urinary tract infection	163 (7.8)	8	93 (9.6)	9	39 (8.2)	49 (12.3)	5 (5.4)	70 (6.2)	8	32 (6.7)	36 (7.7)	2 (1.1)
Peri-prosthetic joint infection	149 (7.1)	20	36 (3.7)	19	4 (0.8)	27 (6.8)	5 (5.4)	113 (10)	20	4 (0.8)	80 (17.2)	29 (15.8)
Superficial wound infection	147 (7)	11	59 (6.1)	11	22 (4.6)	32 (8)	5 (5.4)	88 (7.8)	13	25 (5.2)	58 (12.5)	5 (2.7)
Skin. tissue and superficial vessel harm[1]	123 (5.9)	4	78 (8.1)	5	59 (12.4)	18 (4.5)	1 (1.1)	45 (4)	4	38 (8)	7 (1.5)	0 (0)
Peri-/post-operative bleeding/ haematoma - did not require re-operation	117 (5.6)	0	29 (3)	3	18 (3.8)	9 (2.3)	2 (2.2)	88 (7.8)	0	61 (12.8)	26 (5.6)	1 (0.5)
Pneumonia	117 (5.6)	4	77 (8)	3	47 (9.9)	25 (6.3)	5 (5.4)	40 (3.6)	5	21 (4.4)	13 (2.8)	6 (3.3)
Falls	87 (4.2)	15	38 (3.9)	19	6 (1.3)	22 (5.5)	10 (10.8)	49 (4.4)	10	17 (3.6)	24 (5.2)	8 (4.4)
Neurological[2]	87 (4.2)	2	46 (4.8)	2.5	33 (6.9)	11 (2.8)	2 (2.2)	41 (3.6)	2	30 (6.3)	9 (1.9)	2 (1.1)
Distended urinary bladder	81 (3.9)	3	52 (5.4)	3	39 (8.2)	12 (3)	1 (1.1)	29 (2.6)	3	24 (5)	5 (1.1)	0 (0)
Pulmonary embolism	64 (3.1)	27	15 (1.5)	12	3 (0.6)	8 (2)	4 (4.3)	49 (4.4)	32	9 (1.9)	15 (3.2)	25 (13.7)
Gastric ulcer	45 (2.2)	6	19 (2)	7	8 (1.7)	10 (2.5)	1 (1.1)	26 (2.3)	5.5	12 (2.5)	13 (2.8)	1 (0.5)
Cardiovascular[3]	40 (1.9)	3	14 (1.4)	13.5	6 (1.3)	5 (1.3)	3 (3.2)	26 (2.3)	3	17 (3.6)	7 (1.5)	2 (1.1)
Pain	36 (1.7)	4	7 (0.7)	3	4 (0.8)	3 (0.8)	0 (0)	29 (2.6)	9	13 (2.7)	13 (2.8)	3 (1.6)
Gastro-intestinal[4]	31 (1.5)	7	9 (0.9)	7	4 (0.8)	4 (1)	1 (1.1)	22 (2)	6.5	11 (2.3)	11 (2.4)	0 (0)
Peri-operative fracture	31 (1.5)	0	8 (0.8)	0	6 (1.3)	2 (0.5)	0 (0)	23 (2)	0	20 (4.2)	3 (0.6)	0 (0)
Renal failure	31 (1.5)	2	20 (2.1)	2	18 (3.8)	2 (0.5)	0 (0)	11 (1)	3	10 (2.1)	1 (0.2)	0 (0)
Deep vein thrombosis	29 (1.4)	42	10 (1)	24.5	0 (0)	6 (1.5)	4 (4.3)	19 (1.7)	46	0 (0)	6 (1.3)	13 (7.1)
Allergic reaction	25 (1.2)	5	8 (0.8)	5	4 (0.8)	4 (1)	0 (0)	17 (1.5)	4	11 (2.3)	4 (0.9)	2 (1.1)
Leg length difference	23 (1.1)	3	4 (0.4)	2.5	3 (0.6)	1 (0.3)	0 (0)	19 (1.7)	3	14 (2.9)	3 (0.6)	2 (1.1)

For peer review only

Gastrointestinal infection	22 (1.1)	9	17 (1.8)	11	8 (1.7)	8 (2)	1 (1.1)	5 (0.4)	4	4 (0.8)	1 (0.2)	0 (0)
Septicaemia	20 (1)	17.5	14 (1.4)	17.5	6 (1.3)	7 (1.8)	1 (1.1)	6 (0.5)	13.5	2 (0.4)	3 (0.6)	1 (0.5)
Mechanical complication[5]	20 (1)	25	7 (0.7)	24	3 (0.6)	4 (1)	0 (0)	13 (1.2)	29	2 (0.4)	5 (1.1)	6 (3.3)
Unclear infection	17 (0.8)	5	11 (1.1)	5	5 (1.1)	6 (1.5)	0 (0)	6 (0.5)	4	5 (1)	1 (0.2)	0 (0)
Respiratory	17 (0.8)	1	9 (0.9)	1	6 (1.3)	3 (0.8)	0 (0)	8 (0.7)	1	7 (1.5)	1 (0.2)	0 (0)
Myocardial infarction	15 (0.7)	7	6 (0.6)	3.5	3 (0.6)	3 (0.8)	0 (0)	9 (0.8)	8	5 (1)	3 (0.6)	1 (0.5)
Mouth and throat infection	12 (0.6)	9	8 (0.8)	9	5 (1.1)	3 (0.8)	0 (0)	4 (0.4)	10	2 (0.4)	2 (0.4)	0 (0)
Peripheral nerve injury[6]	12 (0.6)	2	3 (0.3)	8	1 (0.2)	2 (0.5)	0 (0)	9 (0.8)	2	8 (1.7)	1 (0.2)	0 (0)
Ileus	9 (0.4)	5	4 (0.4)	6	0 (0)	4 (1)	0 (0)	5 (0.4)	4	3 (0.6)	2 (0.4)	0 (0)
Stroke	9 (0.4)	4	4 (0.4)	5	3 (0.6)	1 (0.3)	0 (0)	5 (0.4)	4	3 (0.6)	1 (0.2)	1 (0.5)
Electrolyte imbalance	8 (0.4)	5.5	1 (0.1)	5	1 (0.2)	0 (0)	0 (0)	7 (0.6)	6	2 (0.4)	5 (1.1)	0 (0)
AE caused by anaesthesia[7]	7 (0.3)	1	3 (0.3)	0	3 (0.6)	0 (0)	0 (0)	4 (0.4)	1	3 (0.6)	1 (0.2)	0 (0)
Malnutrition	7 (0.3)	6	4 (0.4)	13	2 (0.4)	1 (0.3)	1 (1.1)	3 (0.3)	6	1 (0.2)	2 (0.4)	0 (0)
Bleeding - not related to surgery[8]	5 (0.2)	11	2 (0.2)	6.5	1 (0.2)	1 (0.3)	0 (0)	3 (0.3)	11	0 (0)	3 (0.6)	0 (0)
Infection other	5 (0.2)	11	4 (0.4)	11	1 (0.2)	3 (0.8)	0 (0)	1 (0.1)	6	0 (0)	1 (0.2)	0 (0)
Bleeding - that required re-operation	5 (0.2)	8	1 (0.1)	8	1 (0.2)	0 (0)	0 (0)	4 (0.4)	5	3 (0.6)	1 (0.2)	0 (0)
Surgical harm – other	3 (0.1)	71	0 (0)	NA	– (–)	– (–)	– (–)	3 (0.3)	71	0 (0)	0 (0)	3 (1.6)
Multi-organ failure	1 (0)	3	1 (0.1)	3	1 (0.2)	0 (0)	0 (0)	0 (0)	NA	– (–)	– (–)	– (–)
Other	10 (0.5)	7.5	3 (0.3)	19	1 (0.2)	1 (0.3)	1 (1.1)	7 (0.6)	7	2 (0.4)	4 (0.9)	1 (0.5)
Total	2093 (100)	8	968 (100)	8	476 (100)	399 (100)	93 (100)	1125 (100)	8	477 (100)	465 (100)	183 (100)

AE, adverse event.

Examples of adverse events in these types; [1] blister, extravasation, phlebitis; [2] acute confusion, hallucination, lethargy; [3] heart failure; [4] obstipation, vomiting, diarrhoea; [5] fracture without a fall, re-operation after several dislocations; [6] foot drop; [7] awareness, aspiration; [8] in connection to urinary catheter or warfarin treatment.

For peer review only

STROBE Statement—Checklist of items that should be included in reports of ***cohort studies***

	Item No	Recommendation	Page No
**Title and abstract**	1	(*a*) Indicate the study's design with a commonly used term in the title or the abstract	1
		(*b*) Provide in the abstract an informative and balanced summary of what was done and what was found	2
**Introduction**			
Background/rationale	2	Explain the scientific background and rationale for the investigation being reported	4
Objectives	3	State specific objectives, including any prespecified hypotheses	4
**Methods**			
Study design	4	Present key elements of study design early in the paper	5
Setting	5	Describe the setting, locations, and relevant dates, including periods of recruitment, exposure, follow-up, and data collection	5
Participants	6	(*a*) Give the eligibility criteria, and the sources and methods of selection of participants. Describe methods of follow-up<br>(*b*) For matched studies, give matching criteria and number of exposed and unexposed	5
Variables	7	Clearly define all outcomes, exposures, predictors, potential confounders, and effect modifiers. Give diagnostic criteria, if applicable	6
Data sources/ measurement	8*	For each variable of interest, give sources of data and details of methods of assessment (measurement). Describe comparability of assessment methods if there is more than one group	6-7
Bias	9	Describe any efforts to address potential sources of bias	NA
Study size	10	Explain how the study size was arrived at	5 (references)
Quantitative variables	11	Explain how quantitative variables were handled in the analyses. If applicable, describe which groupings were chosen and why	NA
Statistical methods	12	(*a*) Describe all statistical methods, including those used to control for confounding<br>(*b*) Describe any methods used to examine subgroups and interactions<br>(*c*) Explain how missing data were addressed<br>(*d*) If applicable, explain how loss to follow-up was addressed<br>(*e*) Describe any sensitivity analyses	7
**Results**			
Participants	13*	(a) Report numbers of individuals at each stage of study—eg numbers potentially eligible, examined for eligibility, confirmed eligible, included in the study, completing follow-up, and analysed<br>(b) Give reasons for non-participation at each stage<br>(c) Consider use of a flow diagram	5
Descriptive data	14*	(a) Give characteristics of study participants (eg demographic, clinical, social) and information on exposures and potential confounders<br>(b) Indicate number of participants with missing data for each variable of interest<br>(c) Summarise follow-up time (eg, average and total amount)	8-9
Outcome data	15*	Report numbers of outcome events or summary measures over time	8-13

For peer review only - http://bmjopen.bmj.com/site/about/guidelines.xhtml

Main results	16	(*a*) Give unadjusted estimates and, if applicable, confounder-adjusted estimates and their precision (eg, 95% confidence interval). Make clear which confounders were adjusted for and why they were included<br>(*b*) Report category boundaries when continuous variables were categorized<br>(*c*) If relevant, consider translating estimates of relative risk into absolute risk for a meaningful time period	NA
Other analyses	17	Report other analyses done—eg analyses of subgroups and interactions, and sensitivity analyses	NA
**Discussion**			
Key results	18	Summarise key results with reference to study objectives	14
Limitations	19	Discuss limitations of the study, taking into account sources of potential bias or imprecision. Discuss both direction and magnitude of any potential bias	17
Interpretation	20	Give a cautious overall interpretation of results considering objectives, limitations, multiplicity of analyses, results from similar studies, and other relevant evidence	14-17
Generalisability	21	Discuss the generalisability (external validity) of the study results	18
**Other information**			
Funding	22	Give the source of funding and the role of the funders for the present study and, if applicable, for the original study on which the present article is based	20

*Give information separately for exposed and unexposed groups.

Note: An Explanation and Elaboration article discusses each checklist item and gives methodological background and published examples of transparent reporting. The STROBE checklist is best used in conjunction with this article (freely available on the Web sites of PLoS Medicine at http://www.plosmedicine.org/, Annals of Internal Medicine at http://www.annals.org/, and Epidemiology at http://www.epidem.com/). Information on the STROBE Initiative is available at http://www.strobe-statement.org.

For peer review only
